# Transcriptome profiling of *Hyacinthus orientalis* L. cultivars in floral pigmentation

**DOI:** 10.1038/s41597-025-04977-y

**Published:** 2025-04-24

**Authors:** Kwan-Ho Wong, Hoi-Yan Wu, Cheryl Wood-Yee Shum, Jerome Ho-Lam Hui, Pang-Chui Shaw, David Tai-Wai Lau

**Affiliations:** 1 Shiu-Ying Hu Herbarium, School of Life Sciences, The Chinese University of Hong Kong, Shatin, New Territories, Hong Kong SAR, China; 2 School of Life Sciences, The Chinese University of Hong Kong, Shatin, New Territories, Hong Kong SAR, China; 3 Li Dak Sum Yip Yio Chin R & D Centre for Chinese Medicine, The Chinese University of Hong Kong, Shatin, New Territories, Hong Kong SAR, China; 4 Simon F.S. Li Marine Science Laboratory, Institute of Environment, Energy and Sustainability, State Key Laboratory of Agrobiotechnology, The Chinese University of Hong Kong, Shatin, Hong Kong, China; 5 Institute of Chinese Medicine, The Chinese University of Hong Kong, Shatin, Hong Kong, China

**Keywords:** Secondary metabolism, Plant physiology

## Abstract

Hyacinth (*Hyacinthus orientalis* L.) is a popular floricultural crop. Its cultivars exhibit a wide range of phenotypic variations, especially flower colours. Yet, the cultivar pedigree was poorly recorded, impeding efficient breeding in producing novel cultivars. In addition, scarce genomic resource of the species hinders the exploration on the molecular mechanism in controlling the diversification of floral colour. In this study, transcriptome profiling was conducted on seven hyacinth cultivars, each representing a major flower colour. RNA-Seq libraries were prepared from 189 samples that were collected in three perianth partitions at three consecutive developmental stages in biological triplicates. A total of 1,256.8 gigabytes data were generated. The reproducibility and variability of our dataset were assessed through correlation analysis and principal component analysis, respectively. In addition, the usability of the dataset was demonstrated by the identification of differentially expressed genes, functional annotation and functional enrichment analysis. This study provides the first spatiotemporal profiling of the gene expression of hyacinths, contributing to molecular breeding of hyacinth cultivars with novel flower colours.

## Background & Summary

*Hyacinthus orientalis* L., commonly known as hyacinth or garden hyacinth, is one of the most popular floricultural crops. In Angiosperm Phylogenetic Group (APG) IV system^1^, the species is classified under the family Asparagaecae subfamily Scilloideae, which is signatured by its storage organ as bulb, basal leave arrangement and raceme inflorescence^2^. With strong fragrance and brilliant flower colours, hyacinth is always a frequent candidate for potted ornamentals, cut flowers, floral arrangement and flowerbeds. Hyacinth is native to the eastern Mediterranean including countries like Turkey^3^, Israel^4^, Lebanon^5^, Cyprus^4,6^ and Syria^7,8^. Since its introduction to Europe in 1562^9^, the long-lasting domestication for over 460 years has shaped this plant into diversified phenotypes, including cultivars with distinct flower colours and forms. Double-flower cultivars were once abundant in hyacinth in the 18^th^ century^10^, yet most of them were unable to survive^9^. Nowadays, most of the existing cultivars of hyacinths are single flowered, with flower colours almost covered the spectrum of visible light which can be categorised into 8 classes - red, pink, orange, yellow, white, blue, purple and nearly black. More importantly, flower colour is still one of the major targets for breeding novel cultivar to obtain considerable revenue, as demonstrated by the case of the black-flower cultivar ‘Midnight Mystique’^11^.

The ancestor of modern hyacinth cultivars is far less attractive, as shown in its specimens (lectotype - G. Clifford, BM000558527^12^; syntype - van Royen, L0052779^13^; specimen of wild type – BATMAN 014, Supplementary Figure S1) with only a few bell-shaped flowers in pale blue sparsely arranged on a slender spike. Spontaneous mutation and hybridisation were the major traditional breeding methods for producing new hyacinth cultivars with novel flower colours^14^. The order of new flower colour appearance in hyacinth cultivar was loosely recorded in the domestication history^15^: white (in 1582), purple (in 1596), red (in 1614) and yellow (in 1767). The power of domestication has miraculously turned this monotonous plant into a colourful floricultural crop with economic importance. Particularly, hyacinth hold the balance of power to the floricultural industry of the Netherlands, where contributes 95% worldwide production^16^. Noteworthily, hyacinth is the fifth best-selling bulbous plant in the global flower bulb trade^14^, contributing enormous economic income to the society. Additionally, hyacinth is the only bulbous floricultural crop with cultivars in diversified flower colours sharing a single protospecies as *Hyacinthus orientalis* L. It is contrasting to other famous bulbous ornamentals like tulips^17^, lilies^18^ and daffodils^19^, of which the cultivars were crossbred from a number of species. Unlocking the mechanism underlying the diversification of floral pigments is essentially critical to molecular breeding of bulbous ornamentals, which take years for the first flowering from seeds.

The wide range of flower colours in hyacinth cultivars is contributed by the flavonoids, including anthocyanins and anthoxanthins. The three anthocyanins primarily synthesised in the anthocyanin biosynthetic pathway – cyanidin, pelargonidin and delphinidin^20^ – are known to give magenta-red, orange-red and purplish-blue colour, respectively^21^. Further structural modification results in other existing forms. Peonidin in magenta is derived from cyanidin, while malvidin in purplish-blue and petunidin in dark purple or dark red are derived from delphinidin^20,21^. Anthoxanthins, the collective name of flavones and flavonols, give white to yellow colour or even colourlessness^20^.

The composition of flavonoids varies across hyacinth cultivars. In the red-flower cultivar ‘Hollyhock’, derivatives of pelargonidin and cyanidin were identified^22–24^, while in the blue-flower cultivar ‘Delft Blue’, derivatives of delphinidin, petunidin, cyanidin and pelargonidin were found^25^. A systematic profiling on the floral pigments of twelve hyacinth cultivars was done by Tao *et al*. in 2015^26^. Pelargonidin derivatives were found in the cultivars with pink and orange flowers, whereas both pelargonidin derivatives and cyanidin derivatives were identified in cultivars with red, purple, violet and blue flowers. Flavones exist in the perianths of all studied cultivars, as reflected by the yellowish colouration in 30% ammonia. In 2019, Su *et al*. determined the floral pigments in 27 hyacinth cultivars by observing colour reaction in different reagents^27^. It was found that flavonols exist in the red flowers of ‘Jan Bos’ as 25% ammonia water turned dark yellow. Both Tao’s and Su’s studies revealed that perianths of hyacinth cultivars are free of coloured carotenoids, since no colour change was observed in petroleum ether, regardless of flower colour^26,27^. In addition, no anthocyanin was found in the flowers of the cultivars blossoming in yellow and white.

Application of multi-omics and advance sequencing technologies, including next-generation sequencing (NGS) and the third-generation sequencing (TGS), in studying model plants^28^ and other economically valuable plants^29^ are common. Unfortunately, hyacinth was always out of the list. Till now there is no reference genome at chromosome level assembled for hyacinth, but only an unpublished genome assembled at scaffold-level deposited in GenBank (GCA_031762755.1). Limited genomic resources were available for this understudied plant. Our group contributed the complete plastid genomes of the species in a study exploring phylogenetic relationships of seven cultivars with distinct flower colours^30^. However, these data cannot unlock the molecular mechanism for the diversification of floral pigmentation. More specifically speaking, which genes can affect the biosynthesis of floral pigments? How does the expression (upregulation or downregulation) of these genes contribute to the pigment composition? Would the co-expressions of these differentially expressed genes (DEGs) formulate a particular flower colour? Transcriptomic data could address these research questions and assist the breeders in creating novel hyacinth cultivars with desired and even undiscovered traits.

In 2020, Li and collaborators published the mechanism of flower colour diversification in hyacinth cultivars studied by transcriptomic technologies^31^. They revealed that *HoF3’5’H1* and *HoFOMT2* are key genes involved in peonidin synthesis in a black-flower cultivar, while *HoDFR2* is related to the biosynthesis of pelargonidin in the cultivars with blue and red flowers. The transcription factor *HoMYB5* is a key gene in the suppression of anthocyanin biosynthesis in a white-flower cultivar^31^. However, their transcriptomic dataset was prepared from a single cultivar. We therefore set forth to explore the DEGs between cultivars, developmental stages and floral partitions.

In this study, we applied top-down approach in exploring the underlying genotypes on the seven selected cultivars with different flower colours. This study was designed to provide transcriptomic data which are referrable and valuable to both scientists and breeders to explore the biosynthesis of floral pigments in hyacinth and its closely related ornamentals, contributing to the new generation of molecular breeding.

## Methods

### Plant materials and collection of RNA samples

Seven hyacinth cultivars with distinct flower colours were selected, namely ‘Jan Bos’ (JB) in red, ‘Pink Pearl’ (PP) in pink, ‘Gipsy Queen’ (GQ) in orange, ‘City of Haarlem’ (CH) in yellow, ‘China Pink’ (CP) in pinkish white, ‘Delft Blue’ (DB) in blue and ‘Peter Stuyvesant’ (PS) in dark purple. The bulbs were imported from the Netherlands (Simple Pleasures Flowerbulbs & Perennials Inc.) in September 2022. They were firstly immersed in tetrachlorophthalonitrile for 1 hour to prevent fungal infection. Air-dried bulbs were stored at 4 °C for three months. The vernalised bulbs were planted on 3^rd^ January 2023, in an outdoor environment outside Li Dak Sum Yip Yio Chin R&D Centre for Chinese Medicine (GPS: 22.419292, 114.210200) with natural sunlight. Perianths were collected during 16^th^ January to 4^th^ February 2023.

**Fig. 1:**
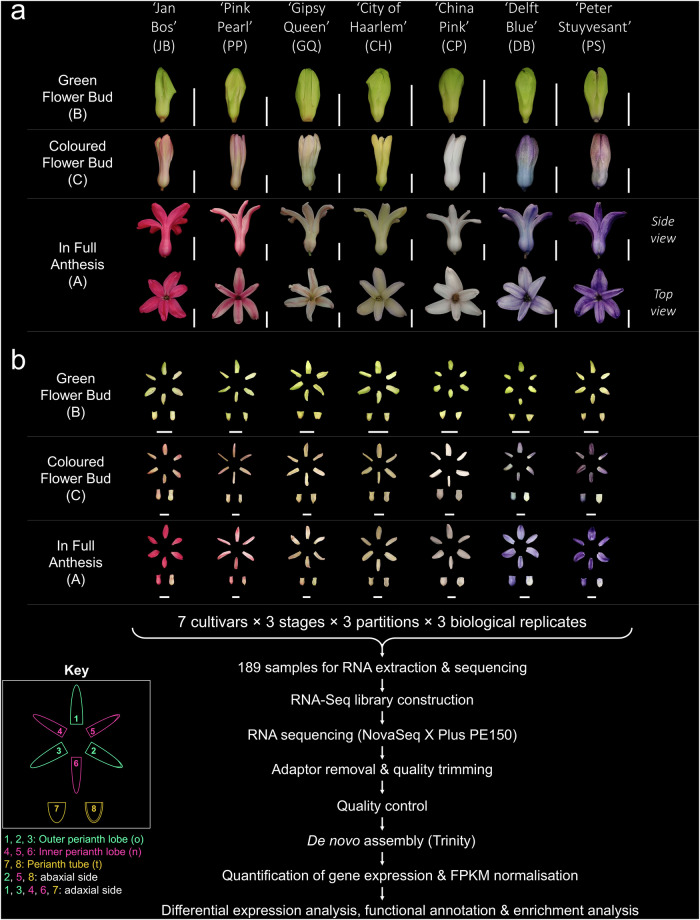
Summary of experiment design, sampling and workflow of the study. (**a**) Three consecutive developmental stages were targeted for the seven studied hyacinth cultivars. The flower buds of all cultivars were initially green at the first stage (B). Changes and differentiation of colour on flower buds were observed at the second stage (C). Flowers were in full anthesis at the third stage (A). The side view and top view of the flower at the third stage were documented for each cultivar, showing the colour differences of each partition. The white scale bar in each photo represents the length of 1 cm. (**b**) For each biological sample at each developmental stage, the perianth was further divided into three partitions, namely the outer perianth lobes (o), the inner perianth lobes (n) and the perianth tube (t). Perianth partitions are numbered as in the key. 1,2,3 - outer perianth lobes; 4,5,6 - inner perianth lobes; 7,8 - perianth tube; 2,5,8 - abaxial side; 1,3,4,6,7 - adaxial side. The white scale bar in each photo represents the length of 1 cm. A total of 189 RNA samples were collected, extracted and sequenced. The RNA-Seq libraries were sequenced on NovaSeq X Plus Platform, with a paired-end read size of 150 nucleotides. Before *de novo* assembly using Trinity, quality control was carried on the raw reads, during which the adaptors and low-quality reads were removed to generate clean reads. After assembly, gene expression levels were first quantified and then normalised into FKPM. Analyses including differential expression analysis, functional annotations and functional enrichment analysis were conducted.

Three consecutive developmental stages were targeted, namely the first stage with green flower buds (B), the second stage with coloured flower buds (C) and the third stage with flowers in full anthesis (A; Fig. 1a). Three perianth partitions of each flower were collected separately, namely the outer perianth lobes (o), the inner perianth lobes (n), and the perianth tube (t; Fig. 1b). The perianth partitions of a single flower were dissected with sterile blade, with removal of androecium and gynoecium using autoclaved forceps. Divided floral partitions were fragmented with sterile blade, then immediately submerged in 1.2 mL RNA*later*™ Stabilization Solution (Invitrogen, MA, USA) and stored overnight at 4 °C allowing full penetration before long-term storage at −20 °C. Three biological replicates were collected for each perianth partitions at different timepoints. A total of 189 samples were collected for RNA sequencing. The voucher specimens of the seven studied cultivars (K. H. Wong 327, 328, 330, 332, 333, 335 & 336; Supplementary Figures S2–S8) were deposited in Shiu-Ying Hu Herbarium (herbarium code: CUHK), School of Life Sciences, the Chinese University of Hong Kong. Absorbent boards (catalogue number PW104) manufactured by Kunming Plantwise Biotech Co., Ltd. (Yunnan, China) were adopted for specimen pressing to retain flower pigments from degradation.

### RNA extraction, sequencing and de novo assembly

Total RNA was extracted using Qiagen RNeasy Plant Mini Kit (Qiagen Co., Hilden, Germany) following the instructions of manufacturer. The quality and quantity of extracted RNA were assessed by 1.5% agarose gel electrophoresis and Nanodrop Lite Spectrophotometer (Thermo Fisher Scientific, MA, USA), respectively. The gel records (Supplementary Figures S9–S19) and readings of spectrophotometer (Supplementary Table S1) were included in the additional information. Sequencing, *de novo* assembly and standard analyses of RNA-Seq libraries were done by Novogene Co., Ltd. (http://en.novogene.com/, Beijing, China). Libraries of mRNA with poly-A enrichment were sequenced on Illumnia NovaSeq X Plus platform, with paired-end sequencing of 150 nucleotides. About 6 GB data were targeted for each sample. A total of 189 RNA-Seq datasets in 1,256.8 GB were generated. Each biological replicate was given a unique sample code. For example, GQ1oA, GQ2oA, GQ3oA are the three biological replicates of the outer perianth lobes of the cultivar ‘Gipsy Queen’ collected at the third stage (flowers in full anthesis). The sample code is equivalent to the library ID of that sample (Table 1).

**Table 1:** Sample code of the 189 samples collected at different developmental stages and partitions in the seven studied cultivars.

Cultivar	Stage	Green Buds			Coloured Buds			Full Anthesis		
	Replicate	Outer lobes	Inner lobes	Perianth tube	Outer lobes	Inner lobes	Perianth tube	Outer lobes	Inner lobes	Perianth tube
‘Jan Bos’	1	JB1oB	JB1nB	JB1tB	JB1oC	JB1nC	JB1tC	JB1oA	JB1nA	JB1tA
	2	JB2oB	JB2nB	JB2tB	JB2oC	JB2nC	JB2tC	JB2oA	JB2nA	JB2tA
	3	JB3oB	JB3nB	JB3tB	JB3oC	JB3nC	JB3tC	JB3oA	JB3nA	JB3tA
‘Pink Pearl’	1	PP1oB	PP1nB	PP1tB	PP1oC	PP1nC	PP1tC	PP1oA	PP1nA	PP1tA
	2	PP2oB	PP2nB	PP2tB	PP2oC	PP2nC	PP2tC	PP2oA	PP2nA	PP2tA
	3	PP3oB	PP3nB	PP3tB	PP3oC	PP3nC	PP3tC	PP3oA	PP3nA	PP3tA
‘Gipsy Queen’	1	GQ1oB	GQ1nB	GQ1tB	GQ1oC	GQ1nC	GQ1tC	GQ1oA	GQ1nA	GQ1tA
	2	GQ2oB	GQ2nB	GQ2tB	GQ2oC	GQ2nC	GQ2tC	GQ2oA	GQ2nA	GQ2tA
	3	GQ3oB	GQ3nB	GQ3tB	GQ3oC	GQ3nC	GQ3tC	GQ3oA	GQ3nA	GQ3tA
‘City of Haarlem’	1	CH1oB	CH1nB	CH1tB	CH1oC	CH1nC	CH1tC	CH1oA	CH1nA	CH1tA
	2	CH2oB	CH2nB	CH2tB	CH2oC	CH2nC	CH2tC	CH2oA	CH2nA	CH2tA
	3	CH3oB	CH3nB	CH3tB	CH3oC	CH3nC	CH3tC	CH3oA	CH3nA	CH3tA
‘China Pink’	1	CP1oB	CP1nB	CP1tB	CP1oC	CP1nC	CP1tC	CP1oA	CP1nA	CP1tA
	2	CP2oB	CP2nB	CP2tB	CP2oC	CP2nC	CP2tC	CP2oA	CP2nA	CP2tA
	3	CP3oB	CP3nB	CP3tB	CP3oC	CP3nC	CP3tC	CP3oA	CP3nA	CP3tA
‘Delft Blue’	1	DB1oB	DB1nB	DB1tB	DB1oC	DB1nC	DB1tC	DB1oA	DB1nA	DB1tA
	2	DB2oB	DB2nB	DB2tB	DB2oC	DB2nC	DB2tC	DB2oA	DB2nA	DB2tA
	3	DB3oB	DB3nB	DB3tB	DB3oC	DB3nC	DB3tC	DB3oA	DB3nA	DB3tA
‘Peter Stuyvesant’	1	PS1oB	PS1nB	PS1tB	PS1oC	PS1nC	PS1tC	PS1oA	PS1nA	PS1tA
	2	PS2oB	PS2nB	PS2tB	PS2oC	PS2nC	PS2tC	PS2oA	PS2nA	PS2tA
	3	PS3oB	PS3nB	PS3tB	PS3oC	PS3nC	PS3tC	PS3oA	PS3nA	PS3tA

**Fig. 2:**
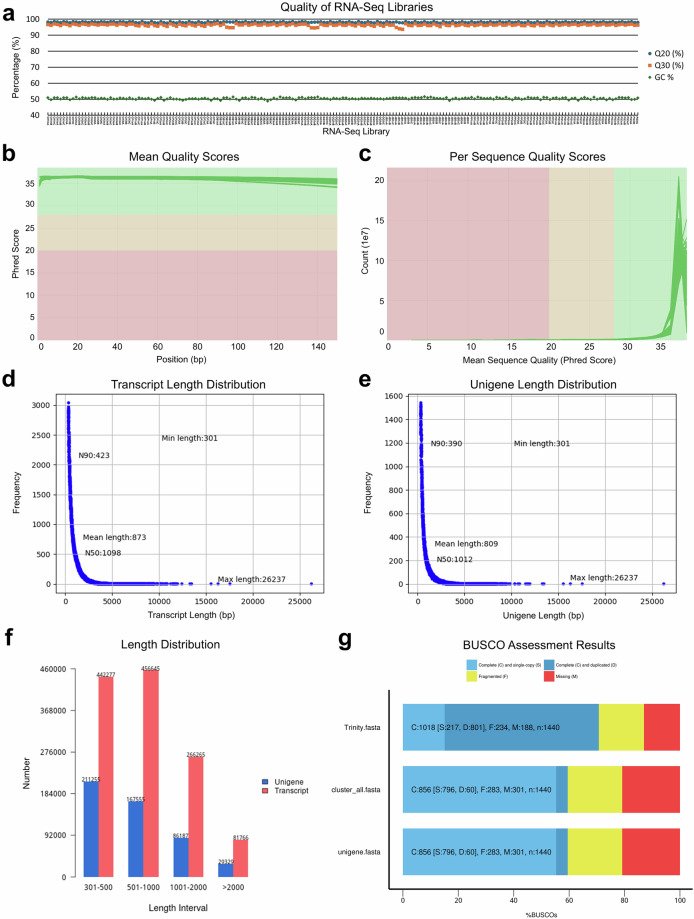
Quality control of sequencing reads and *de novo* assembly. Quality assessment of RNA sequencing and *de novo* assembly. (**a**) The qualities of the 189 transcriptome libraries reflected by the values of Q20, Q30 and GC content. (**b**) Mean quality score per position of the bidirectional reads of the 189 libraries. (**c**) Quality score distribution over the bidirectional reads of all libraries. (**d**) The frequency of transcripts at different lengths. (**e**) The frequency of unigenes at different lengths. (**f**) The length distribution of assembled transcripts and unigenes categorised into different length intervals. (**g**) The results of BUSCO assessment on the completeness of assembled transcripts in a whole dataset (Trinity.fasta), non-redundant transcripts (cluster_all.fasta) and also the longest sequence of each cluster (unigene.fasta).

The raw reads were cleaned by removing the adaptor sequences, the reads with over 0.1% undetermined nucleotides and the reads with over 50 bases showing quality value less than or equal to 5. The Q20, Q30 and GC content of each RNA library are visualised in Fig. 2a. The Q20 of all libraries were above 97%, with only a few outliers showing Q30 lower than 95%. The GC contents of all libraries ranged from 48.85% to 51.55%, with an average value of 50.47%. Meanwhile, the error rate of all libraries was mostly controlled at 0.02% but not exceeding 0.03%. The quality control data of the RNA sequencing is listed in Supplementary Table S2. In addition, the quality of the bidirectional reads for each library were assessed using the software FastQC (https://www.bioinformatics.babraham.ac.uk/projects/fastqc/), and the software multiQC^32^ were adopted to summarise the FastQC reports. The mean quality scores per nucleotide position of all sequencing reads are above the Phred score of 34.00 (Fig. 2b), and most of the sequences are in high quality reflected by the majority scored above 35.00 in Phred score (Fig. 2c). These figures support the high accuracy of the sequencing data.

The cleaned reads were *de novo* assembled into transcripts using the software Trinity^33^. The software CORSET^34^ was adopted to remove the redundant transcripts, through the method of hierarchical clustering based on the proportion of shared reads and expressions. The longest transcript of each cluster was selected as the unigene. The results and quality of *de novo* assembly are visualised in Fig. 2d–f. The maximum length of both transcript and unigene were in 26,237 bp, and the minimum length of both were in 301 bp (Fig. 2d,e). The mean lengths of transcript and unigene were 873 bp and 809 bp, respectively. The N50s of transcript and unigene were 1,098 bp and 1,012 bp, respectively. The largest portion of transcript (36.62%) was in the range of 501–1,000 bp, while the largest portion of unigene (42.74%) fell in the interval of 301–500 bp (Fig. 2f). To evaluate the completeness of the transcript, BUSCO assessment^35^ was done on the whole transcriptome dataset, transcript clusters and unigenes. Before hierarchical clustering, the whole dataset has 70.7% complete transcripts, including 15.1% single and 55.6% duplicated transcripts (Fig. 2g). After clustering, both the cluster and unigene datasets show the same BUSCO values, i.e. 59.5% complete transcripts consisting of 55.3% single and 4.2% duplicated transcripts. There remained 19.7% and 20.8% transcripts that were fragmented and missing, respectively, in the cluster and unigene datasets.

### Quantification and normalisation of gene expression

To quantify the expression level, the software RNA-Seq by Expectation Maximization (RSEM)^36^ was adopted to map the reads to a set of reference transcript sequences filtered by CORSET. The read count as the relative abundance of unigene was calculated based on the alignment of reads against the reference transcript using Bowtie^37^. Then, the read counts were normalised into Fragments Per Kilobase of transcript sequence per Millions (FPKM) value, which considers the effects of both sequencing depth and gene length^38^.

### Specifying colour using RHS Colour Chart

The flower colours of each hyacinth cultivar at Stage A were specified using the Royal Horticultural Society (RHS) Colour Chart (Sixth Edition 2019 reprint). The colour of five divisions, namely the midrib on adaxial side of perianth lobes (I), the periphery on adaxial side of perianth lobes (II), the midrib on abaxial side of perianth lobes (III), the periphery on abaxial side of perianth lobes (IV), and the surface of perianth tube (V), were documented. For each cultivar, 10 flowers in full anthesis (Stage A) were collected for measuring colour, and the most frequent colour was adopted^26^. The results are displayed in Table 2, and the measurement records are attached as Supplementary Table S3.

**Table 2:** Colours of five perianth divisions at Stage A specified by RHS Colour Chart (6^th^ Edition).

Cultivar	Division I	Division II	Division III	Division IV	Division V
	Midrib on adaxial perianth lobes	Periphery on adaxial perianth lobes	Midrib on abaxial perianth lobes	Periphery on abaxial perianth lobes	Surface of perianth tube
‘Jan Bos’	61B - Strong Purplish Red	61B - Strong Purplish Red	59 C - Moderate Purplish Red	59 C - Moderate Purplish Red	59 C - Moderate Purplish Red
‘Pink Pearl’	67B - Vivid Purplish Red	65B - Light Purplish Pink	63B - Strong Purplish Red	62B - Moderate Purplish Pink	67 C - Deep Purplish Pink
‘Gipsy Queen’	35D - Moderate Pink	N170D - Moderate Yellowish Pink	35 C - Strong Yellowish Pink	N170D - Moderate Yellowish Pink	29 C - Light Yellowish Pink & 35D- Moderate Pink
‘City of Haarlem’	11 C - Pale Yellow	11D - Pale Yellow	12 C - Light Yellow	13D - Pale Greenish Yellow	13D - Pale Greenish Yellow
‘China Pink’	69 A - Very Pale Purple	69D - Very Pale Purple	56 A - Pale Purplish Pink	56D - Pale Purplish Pink & 69D - Very Pale Purple	64D - Deep Purplish Pink, 69 C - Very Pale Purple & 56D - Pale Purplish Pink
‘Delft Blue’	97 A - Brilliant Purplish Blue	92 A - Brilliant Violet	98B - Moderate Blue	94B - Strong Purplish Blue	98B - Moderate Blue
‘Peter Stuyvesant’	N88A - Strong Violet	N88B - Strong Violet	N88A - Strong Violet	90B - Strong Violet	94B - Strong Purplish Blue

### Extraction of anthocyanins and flavonoids

The flowers in full anthesis (Stage A) of each cultivar were frozen in liquid nitrogen and stored at −80 °C until being used. About 1 g of perianth were ground into fine powder with liquid nitrogen, then extracted with 2 mL anthocyanin extraction buffer (AEB, Methanol:Water:Formic acid:Trifluoroacetic acid in 70∶27∶2∶1, by volume) for 24 hours under dark^39^. The lysates were centrifuged at maximum speed (15,000 rpm) for phase separation. The supernatants were collected and passed through a 0.45 μm filter membrane. Three biological replicates of each cultivar were extracted. Photo documentation and summary of the extracts are recorded in Supplementary Figure S21 and Supplementary Table S4, respectively. The HPLC-graded standards of anthocyanins (pelargonidin 3-O-glucoside chloride, Pg3O, cyanidin-3-O-glucoside chloride, Cy3O and delphinidin-3-O-glucoside chloride, Dp3O) and flavonoids (kaempferol, quercetin and myricetin) were purchased from Shanghai Yuanye Bio-Technology Co., Ltd (Shanghai, China).

## Data Records

The data underlying this article are available in the National Center for Biotechnology Information (NCBI) Sequence Read Archive (SRA) database under the accession number SRP481794^40^. Each independent RNA-Seq library are under the accession number of SRR27431542 to SRR27431691 and SRR27433589 to SRR27433627 (Table 3). The gene expression data derived from our original transcriptomic datasets are now deposited in NCBI Gene Expression Omnibus (GEO) under the accession number GSE286406^41^. The results of *de novo* assembly, quantification of gene expression level, differential expression analysis, functional annotation, functional enrichment analysis, CDS prediction, detection of SSR, SNP and InDels, together with the supplementary files, were deposited in CUHK Research Data Repository (https://researchdata.cuhk.edu.hk/dataverse/hyacinth_transcriptome)^42^ and available to the public.

**Table 3:** SRA accession numbers corresponding to each biological replicate at different developmental stages and partitions of the seven studied cultivars.

Cultivar	Stage	Green Buds			Coloured Buds			Full Anthesis		
	Replicate	Outer lobes	Inner lobes	Perianth tube	Outer lobes	Inner lobes	Perianth tube	Outer lobes	Inner lobes	Perianth tube
‘**Jan Bos**’	1	SRR27431675	SRR27431679	SRR27431672	SRR27431674	SRR27431677	SRR27431671	SRR27431676	SRR27431680	SRR27431673
	2	SRR27431665	SRR27431669	SRR27431662	SRR27431664	SRR27431668	SRR27431661	SRR27431666	SRR27431670	SRR27431663
	3	SRR27431655	SRR27431659	SRR27431652	SRR27431654	SRR27431658	SRR27431651	SRR27431657	SRR27431660	SRR27431653
‘**Pink Pearl**’	1	SRR27431646	SRR27431649	SRR27431642	SRR27431644	SRR27431648	SRR27431641	SRR27431647	SRR27431650	SRR27431643
	2	SRR27431636	SRR27431639	SRR27433626	SRR27431635	SRR27431638	SRR27433615	SRR27431637	SRR27431640	SRR27433627
	3	SRR27433591	SRR27433594	SRR27433625	SRR27433590	SRR27433593	SRR27433624	SRR27433592	SRR27433604	SRR27433589
‘**Gipsy Queen**’	1	SRR27431557	SRR27431560	SRR27431554	SRR27431556	SRR27431559	SRR27431553	SRR27431558	SRR27431561	SRR27431555
	2	SRR27431547	SRR27431550	SRR27431544	SRR27431546	SRR27431549	SRR27431543	SRR27431548	SRR27431551	SRR27431545
	3	SRR27431685	SRR27431688	SRR27431682	SRR27431684	SRR27431687	SRR27431681	SRR27431686	SRR27431542	SRR27431683
‘**City of Haarlem**’	1	SRR27431607	SRR27431690	SRR27431574	SRR27431596	SRR27431629	SRR27431563	SRR27431618	SRR27431691	SRR27431585
	2	SRR27431656	SRR27431689	SRR27431633	SRR27431645	SRR27431678	SRR27431632	SRR27431667	SRR27431552	SRR27431634
	3	SRR27431626	SRR27431630	SRR27431623	SRR27431625	SRR27431628	SRR27431622	SRR27431627	SRR27431631	SRR27431624
‘**China Pink**’	1	SRR27431616	SRR27431620	SRR27431613	SRR27431615	SRR27431619	SRR27431612	SRR27431617	SRR27431621	SRR27431614
	2	SRR27431606	SRR27431610	SRR27431603	SRR27431605	SRR27431609	SRR27431602	SRR27431608	SRR27431611	SRR27431604
	3	SRR27431597	SRR27431600	SRR27431593	SRR27431595	SRR27431599	SRR27431592	SRR27431598	SRR27431601	SRR27431594
‘**Delft Blue**’	1	SRR27431587	SRR27431590	SRR27431583	SRR27431586	SRR27431589	SRR27431582	SRR27431588	SRR27431591	SRR27431584
	2	SRR27431577	SRR27431580	SRR27431573	SRR27431576	SRR27431579	SRR27431572	SRR27431578	SRR27431581	SRR27431575
	3	SRR27431567	SRR27431570	SRR27431564	SRR27431566	SRR27431569	SRR27431562	SRR27431568	SRR27431571	SRR27431565
‘**Peter Stuyvesant**’	1	SRR27433619	SRR27433622	SRR27433616	SRR27433618	SRR27433621	SRR27433614	SRR27433620	SRR27433623	SRR27433617
	2	SRR27433609	SRR27433612	SRR27433606	SRR27433608	SRR27433611	SRR27433605	SRR27433610	SRR27433613	SRR27433607
	3	SRR27433599	SRR27433602	SRR27433596	SRR27433598	SRR27433601	SRR27433595	SRR27433600	SRR27433603	SRR27433597

## Technical Validation

**Fig. 3:**
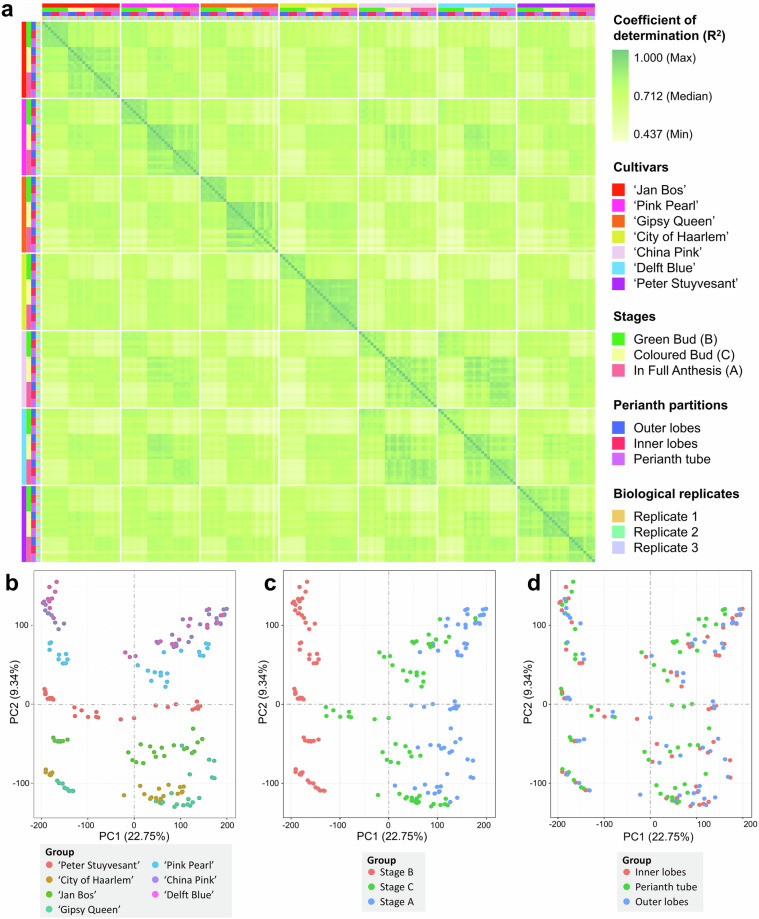
Results of correlation analysis and principal component analysis. (**a**) Correlation plot of the 189 RNA-Seq libraries. The columns and rows are equivalent, under the sequential order of cultivars, developmental stages, perianth partitions and biological replicates. (**b**–**d**) PCA plots of the 189 transcriptome libraries in 2 dimensions, grouped in (**b**) cultivars, (**c**) developmental stages and (**d**) partitions of perianth.

Correlation analysis was conducted to assess the reproducibility of dataset. As shown in Fig. 3a, the biological replicates of each cultivar at different developmental stages and perianth partitions were closely related to each other (coefficient of determination R^2^ close to 1), reflecting high similarities between the replicates. In addition, similarities were also observed between developmental stages and cultivars. Except ‘Peter Stuyvesant’, the replicates of Stage C and Stage A for all other cultivars showed a stronger correlation. For ‘Peter Stuyvesant’, the correlation between the replicates of Stage C and Stage B and was much stronger than that between the replicates of Stage C and Stage A. Meanwhile, the replicates of ‘China Pink’ and ‘Delft Blue’ also showed similar pattern in the correlation plot, which may imply their similarity in gene expression, and further, their phylogenetic relationship.

Principal component analysis (PCA) was conducted to evaluate the variability of sequencing libraries influenced by different factors. The influences from cultivars and developmental stages were observed. As shown in Fig. 3b, clustering of libraries from different cultivars was particularly obviously on the y-axis (PC2), forming distinct layers for almost all cultivars, except ‘China Pink’ and ‘Delft Blue’ which were clustered together. It could be explained by the smaller genetical difference between the two than that between other cultivars, since ‘China Pink’ is the sport (mutant) of ‘Delft Blue’^43^. Developmental stages also influenced the grouping of sequencing libraries on the x-axis (PC1), forming two distinct pillars (Fig. 3c). Some libraries of Stages C and A were clustered together, yet their differences were still observed by their condensation of majorities. Perianth partitions induced no influence on the grouping, as the sequencing libraries of all three partitions clustered together (Fig. 3d).

**Fig. 4:**
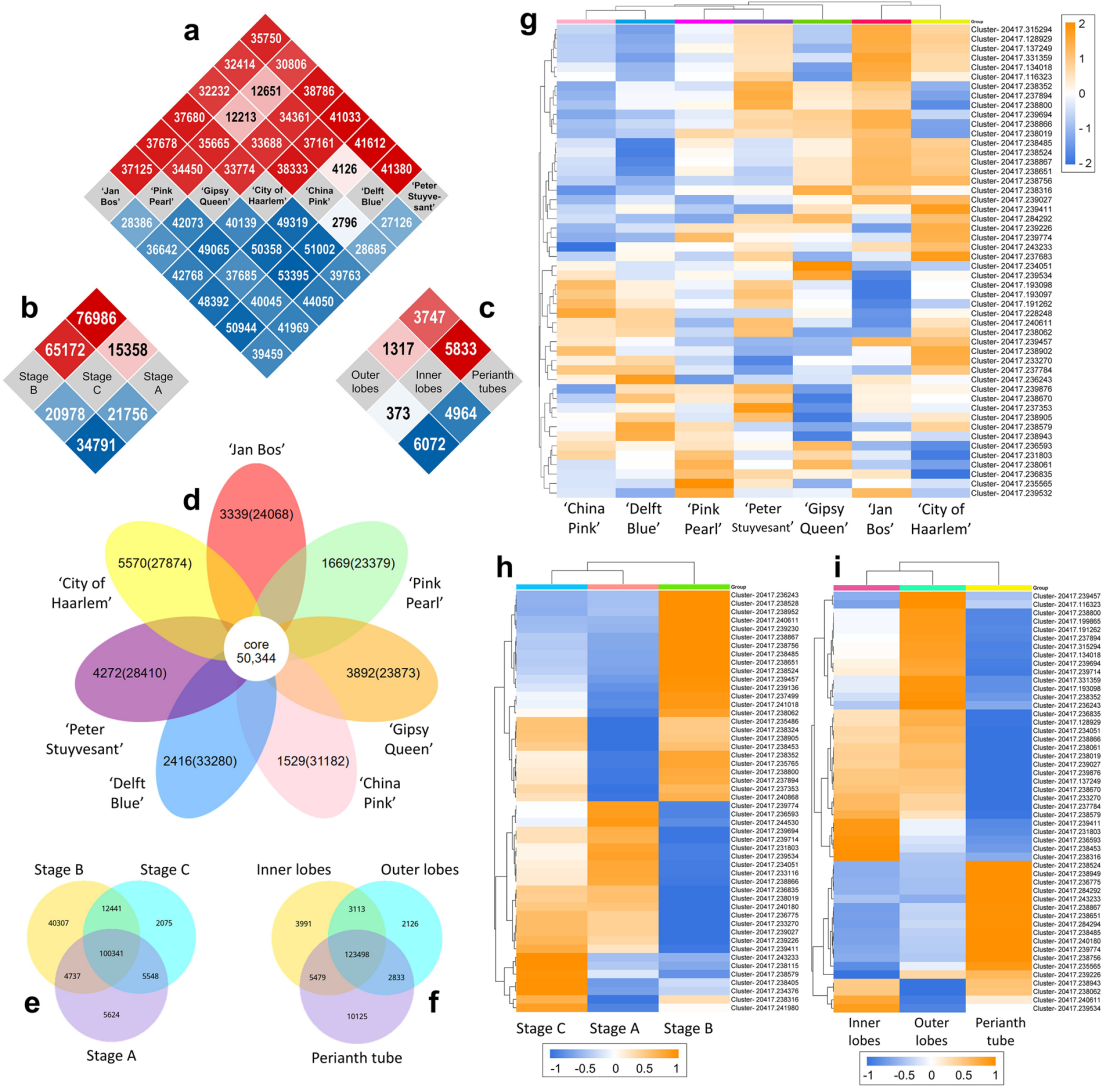
Results of differential expression analysis. Visualisation of DEGs across different parameters. (**a**–**c**) Heatmaps showing the DEGs across different comparisons. Boxes filled in red represent the number of upregulated genes and those filled in blue represent the number of downregulated genes. The numbers in the boxes represent the number of DEGs across the comparison of (**a**) different cultivars, (**b**) different developmental stages and (**c**) different partitions of perianth. (**d**) Flower diagram showing the unique and shared DEGs (as core genes) of the seven studied cultivars. The numbers in the brackets indicate the total number of the transcriptome datasets of that cultivar. (**e,f**) Venn diagrams showing the number of unique (non-overlapped regions) and shared DEGs (overlapped regions) between (**e**) developmental stages and (**f**) perianth partitions. (**g**–**i**) Heatmap visualising the FPKM cluster analysis of DEGs across different parameters, using the log_10_(FPKM + 1) value. Boxes in orange represent upregulated DEGs and those in blue represent downregulaed DEGs. The expression patterns of the selected top 50 DEGs are visualised in (**g**) cultivars, (**h**) developmental stages and (**i**) perianth partitions.

Transcriptomic analyses, including differential expression analysis, functional annotation and functional enrichment analysis, were performed to demonstrate the usability of our dataset while investigating gene expression profiles of hyacinth. The read counts were normalised using DESeq 2^44^ with negative binomial distribution p-value estimation model. The adjusted p-values (padj) were estimated using BH procedure^45^. The DEGs were firstly screened through the standard |log2(FoldChange)| > 1 and padj <0.05. To further filter DEGs across different comparison groups, the threshold DESeq 2 p-value ≤ 0.05 & |log2FoldChange| ≥ 1.0 were adopted. The DEGs identified across different factors, i.e. cultivars, developmental stages and perianth partitions, are visualised in Fig. 4.

The numbers of upregulated and downregulated DEGs identified in each comparison group are visualised in the heatmaps (Fig. 4a–c). Comparing the cultivars, the smallest number of upregulated (4,126) and downregulated genes (2,796) were found in the same comparison group as ‘China Pink’ versus ‘Delft Blue’ (Fig. 4a). The greatest numbers of upregulated (41,612) and downregulated genes (53,395) were observed in two distinct comparison groups, as ‘China Pink’ versus ‘Peter Stuyvesant’ and ‘Gipsy Queen’ versus ‘Delft Blue’, respectively. Comparing the developmental stages, the greatest numbers of upregulated (76,986) and downregulated (34,791) genes were identified in the comparison group Stage B versus Stage A (Fig. 4b). Comparing the perianth partitions, the differential expression between the outer and inner perianth lobes was the smallest (1,317 upregulated and 373 downregulated genes; Fig. 4c).

The number of shared (core) and unique DEGs across the cultivars are visualised in the flower diagram (Fig. 4d). The seven cultivars shared a total of 50,344 DEGs. ‘City of Haarlem’ holds the greatest number of unique DEGs (5,570), while ‘China Pink’ has the smallest number of unique DEGs (1,529). The Venn diagrams (Fig. 4e,f) show the number of shared and unique DEGs identified between different developmental stages and perianth partitions. A total of 100,341 DEGs were found common across all three developmental stages (Fig. 4e). Stage B showed the greatest number of unique DEGs (40,307), while Stage C had the least unique DEGs (2,075). These two stages shared 12,441 DEGs. The commonly shared DEGs in all three perianth partitions accounted for 123,498, and the greatest number of unique DEGs was found in perianth tube (10,125; Fig. 4f).

The expression patterns of the top 50 DEGs across different factors are visualised in the heatmaps (Fig. 4g–i) generated by SRplot^46^. The read counts in FPKM were firstly normalised into the value of log_10_(FPKM + 1). The 50 candidate DEGs in which had significant expression levels were then selected based on the thresholds, i.e. log_10_(FPKM + 1) ≥3.10 for cultivars, ≥3.00 for developmental stages and ≥3.28 for perianth partitions. The DEGs identified across the cultivars at different developmental stages are visualised in the heatmaps in Supplementary Figure S20. These findings facilitate the identification of candidate genes strongly associated with each factor for future validation.

**Fig. 5:**
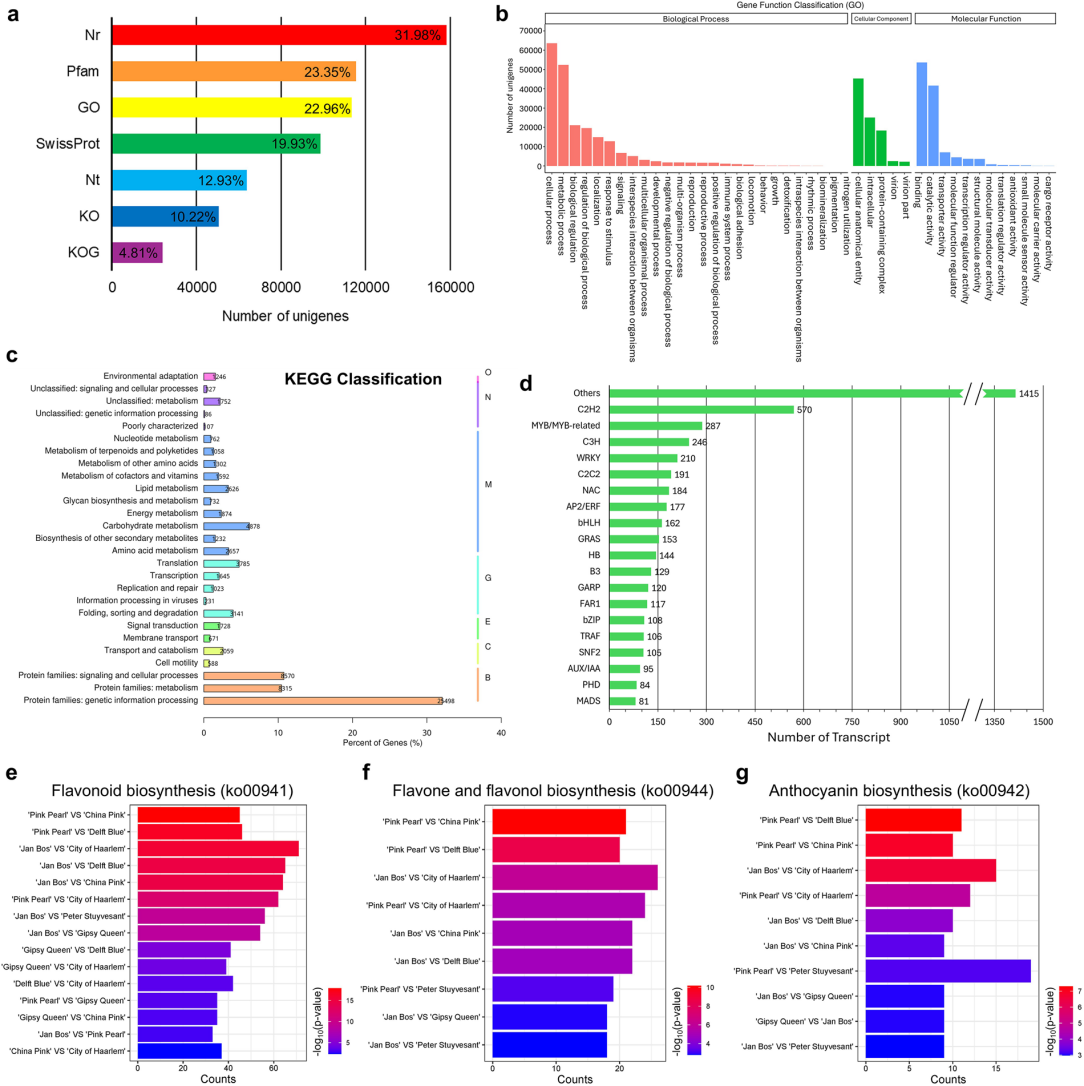
Results of functional annotation and functional enrichment analysis. (**a**–**c**) Functional annotation across different databases. (**a**) The unigenes of all transcriptome datasets were annotated by the seven databases. The x-axis represents the databases, and the y-axis represents the number of unigenes annotated by the respective databases, with the percentages of all unigenes showing on the bars. (**b**) The distribution of GO terms for biological processes (orange-red), cellular components (green) and molecular functions (blue). (**c**) The percentage of genes annotated under KEGG classification. The letters next to the bar lines on the right indicate Level 1 of the KO Pathway. “O” represents Organismal Systems, “N” represents Not Included in Pathway or Brite, “M” represents Metabolism, “G” represents Genetic Information Processing, “E” represents Environmental Information Processing, “C” represents Cellular Processes and “B” represents Brite Hierarchies. (**d**) The number of transcripts annotated under different classes of transcription factors. (**e**–**g**) Through functional enrichment analysis, upregulated expressions were identified in the genes involved in the biosynthesis of (**e**) flavonoid, (**f**) flavone and flavonol, and (**g**) anthocyanin in cultivars across different comparison groups. The gradient in the colour of the bar indicates -log_10_ (p-value); the bar length represents the count of upregulation.

Seven databases were employed to predict gene function, namely Gene Ontology (GO), Kyoto Encyclopedia of Genes and Genome Orthology (KO), euKaryotic Orthologous Groups (KOG), NCBI non-redundant protein sequences (Nr), NCBI nucleotide sequences (Nt), Protein family (Pfam) and Swiss-Prot (Fig. 5a). The annotated unigenes vary across the database, ranging from 23,809 (KOG) to 158,125 (Nr), representing 4.81% and 31.98% of unigenes, respectively. Under the classification of GO, the top three terms for biological processes were “cellular process”, “metabolic process” and “biological regulation”, for cellular component were “cellular anatomical entity”, “intracellular” and “protein-containing complex”, and for molecular functions were “binding”, “catalytic activity” and “transporter activity” (Fig. 5b). Over half of the genes were related to the protein families under KEGG classification and were involved in genetic information processing (32.08%), signalling and cellular processes (10.78%), and metabolism (10.46%; Fig. 5c).

A total of 4,684 transcription factors (TF) were identified using iTAK^47^ and hmmscan (www.hmmer.org). They were classified under different TF families (Fig. 5d). The largest family was C2H2 (570), followed by MYB or MYB-related (287) and C3H (246). TFs involved in floral pigments biosynthesis, including MYB or MYB-related^31,48,49^ (287), bHLH^50^ (162), bZIP^50^ (108) and MADS^51^ (81), were recovered in the TF analysis.

To verify the presence of DEGs involved in floral pigments biosynthesis, functional enrichment analysis was performed with the aid of KEGG database. Upregulation of DEGs were identified between some studied cultivars, and the results are visualised in enrichment bar charts generated by SRplot^46^ (Fig. 5e–g). The comparison group ‘Jan Bos’ versus ‘City of Haarlem’ has the greatest count of upregulated DEGs in flavonoid biosynthesis (71; Fig. 5e) and anthoxanthin biosynthesis (26; Fig. 5f), and the second greatest count of upregulated DEGs in anthocyanin biosynthesis (15; Fig. 5g).

The above outcome of the analyses indicate that our dataset is highly valuable for the study of floral pigments biosynthesis in hyacinth cultivars and is supported by accurate sequencing, reproducible experimental design and reliable data. The molecular mechanism behind floral pigment diversification in hyacinth cultivars can be revealed by using this dataset, which thereby contributes to the molecular breeding of this floricultural crop with high aesthetic and economical value.

## Supplementary information

Supplementary Information

## Data Availability

The software and their versions are described in Methods. No custom code was used in this study.
