# Supplementary material for: Transcriptome profiling of Hyacinthus orientalis L. cultivars in floral pigmentation
Source: Sci Data. 2025 Apr 24;12:689. doi: 10.1038/s41597-025-04977-y (PMC12022083; doi:10.1038/s41597-025-04977-y)
Supplement: Supplementary file 1 — Supplementary Information [file 41597_2025_4977_MOESM1_ESM.pdf]

Transcriptome profiling of *Hyacinthus orientalis* L. cultivars in floral pigmentation

Supplementary Information

Kwan-Ho Wong [1,2,3], Hoi-Yan Wu [3], Cheryl Wood-Yee Shum [1], Jerome Ho-Lam Hui [2,3,4], Pang-Chui Shaw [2,3,5] *, & David Tai-Wai Lau [1,3] *

[1] Shiu-Ying Hu Herbarium, School of Life Sciences, The Chinese University of Hong Kong, Shatin, New Territories, Hong Kong SAR, China.

[2] School of Life Sciences, The Chinese University of Hong Kong, Shatin, New Territories, Hong Kong SAR, China.

[3] Li Dak Sum Yip Yio Chin R & D Centre for Chinese Medicine, The Chinese University of Hong Kong, Shatin, New Territories, Hong Kong SAR, China.

[4] Simon F.S. Li Marine Science Laboratory, Institute of Environment, Energy and Sustainability, State Key Laboratory of Agrobiotechnology, The Chinese University of Hong Kong, Shatin, Hong Kong, China.

[5] Institute of Chinese Medicine and State Key Laboratory of Research on Bioactivities and Clinical Applications of Medicinal Plants (The Chinese University of Hong Kong), The Chinese University of Hong Kong, Shatin, Hong Kong, China.

[*] Correspondences: Pang-Chui Shaw (pcshaw@cuhk.edu.hk); David Tai-Wai Lau (lautaiwai@cuhk.edu.hk)

Table of Content

Fig. S1 – Voucher specimen of wild-type *Hyacinthus orientalis* L. (BATMAN 014) 2
Fig. S2 – Voucher specimen of *Hyacinthus orientalis* L. 'Jan Bos' (K. H. Wong 332).......... 3
Fig. S3 – Voucher specimen of *Hyacinthus orientalis* L. 'Pink Pearl' (K. H. Wong 328).......... 4
Fig. S4 – Voucher specimen of *Hyacinthus orientalis* L. 'Gipsy Queen' (K. H. Wong 327) 5
Fig. S5 – Voucher specimen of *Hyacinthus orientalis* L. 'City of Haarlem' (K. H. Wong 336) 6
Fig. S6 – Voucher specimen of *Hyacinthus orientalis* L. 'China Pink' (K. H. Wong 333) 7
Fig. S7 – Voucher specimen of *Hyacinthus orientalis* L. 'Delft Blue' (K. H. Wong 330) 8
Fig. S8 – Voucher specimen of *Hyacinthus orientalis* L. 'Peter Stuyvesant' (K. H. Wong 335) 9
Fig. S9 – Gel record of total RNA extracted from Stage B perianth partitions 10
Fig. S10 – Gel record of total RNA extracted from Stage B perianth partitions (Cont') 11
Fig. S11 – Gel record of total RNA extracted from Stage B perianth partitions (Cont').......... 12
Fig. S12 – Gel record of total RNA extracted from Stage B perianth partitions (Cont') 13
Fig. S13 – Gel record of total RNA extracted from Stage B perianth partitions (Cont') 14
Fig. S14 – Gel record of total RNA extracted from Stage C perianth partitions 15
Fig. S15 – Gel record of total RNA extracted from Stage C perianth partitions (Cont') 16
Fig. S16 – Gel record of total RNA extracted from Stage C perianth partitions (Cont') 17
Fig. S17 – Gel record of total RNA extracted from Stage A perianth partitions 18
Fig. S18 – Gel record of total RNA extracted from Stage A perianth partitions (Cont').......... 19
Fig. S19 – Gel record of total RNA extracted from Stage A perianth partitions (Cont').......... 20
Fig. S20 – Heatmap visualisation on the top 50 DEGs across the seven cultivars at different developmental stages 21
Fig. S21 – Photo documentation of anthocyanins and flavonoids extracts of the seven cultivars 22

Fig. S1 – Voucher specimen of wild-type *Hyacinthus orientalis* L. (BATMAN 014)

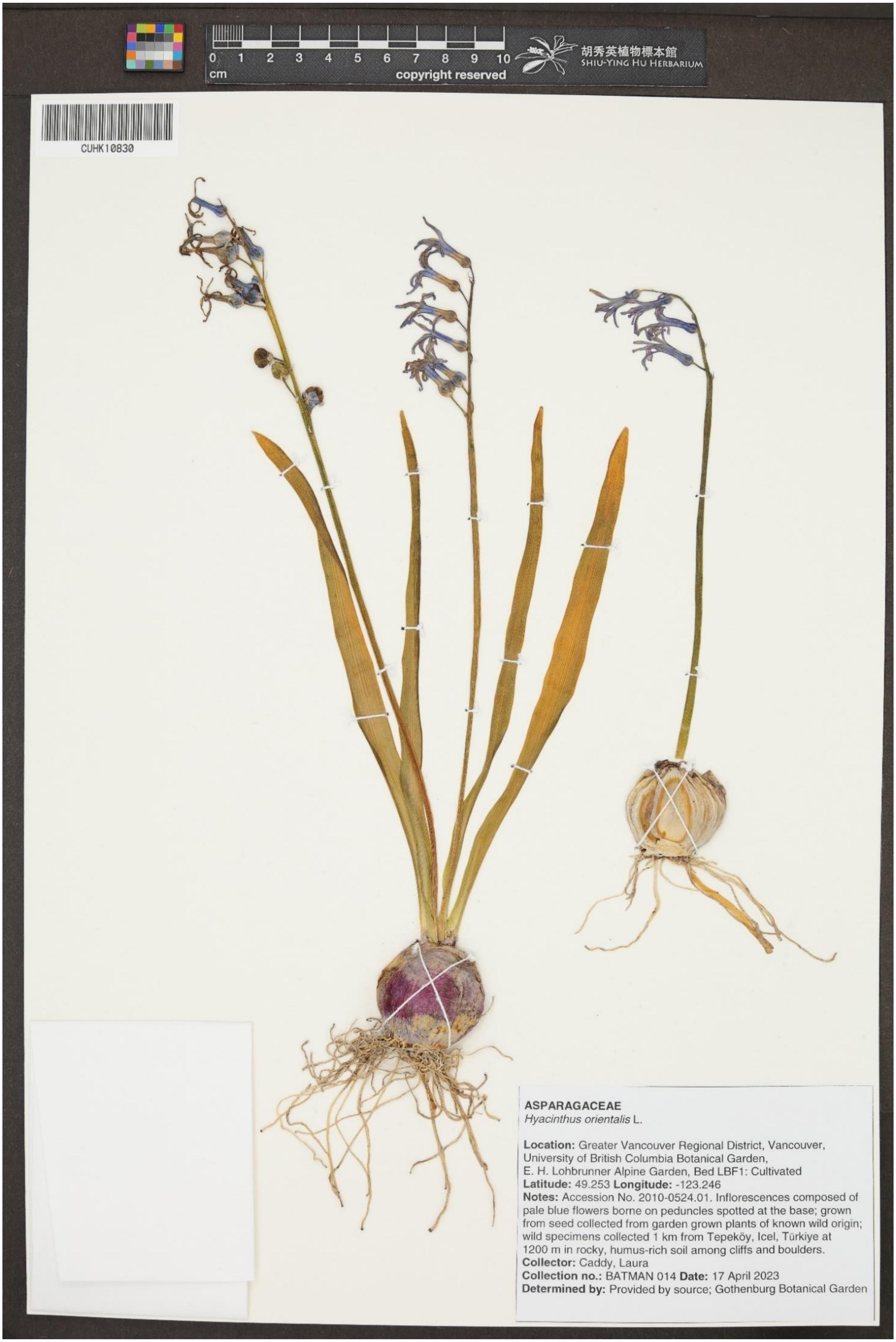

Fig. S2 – Voucher specimen of *Hyacinthus orientalis* L. ‘Jan Bos’ (K. H. Wong 332)

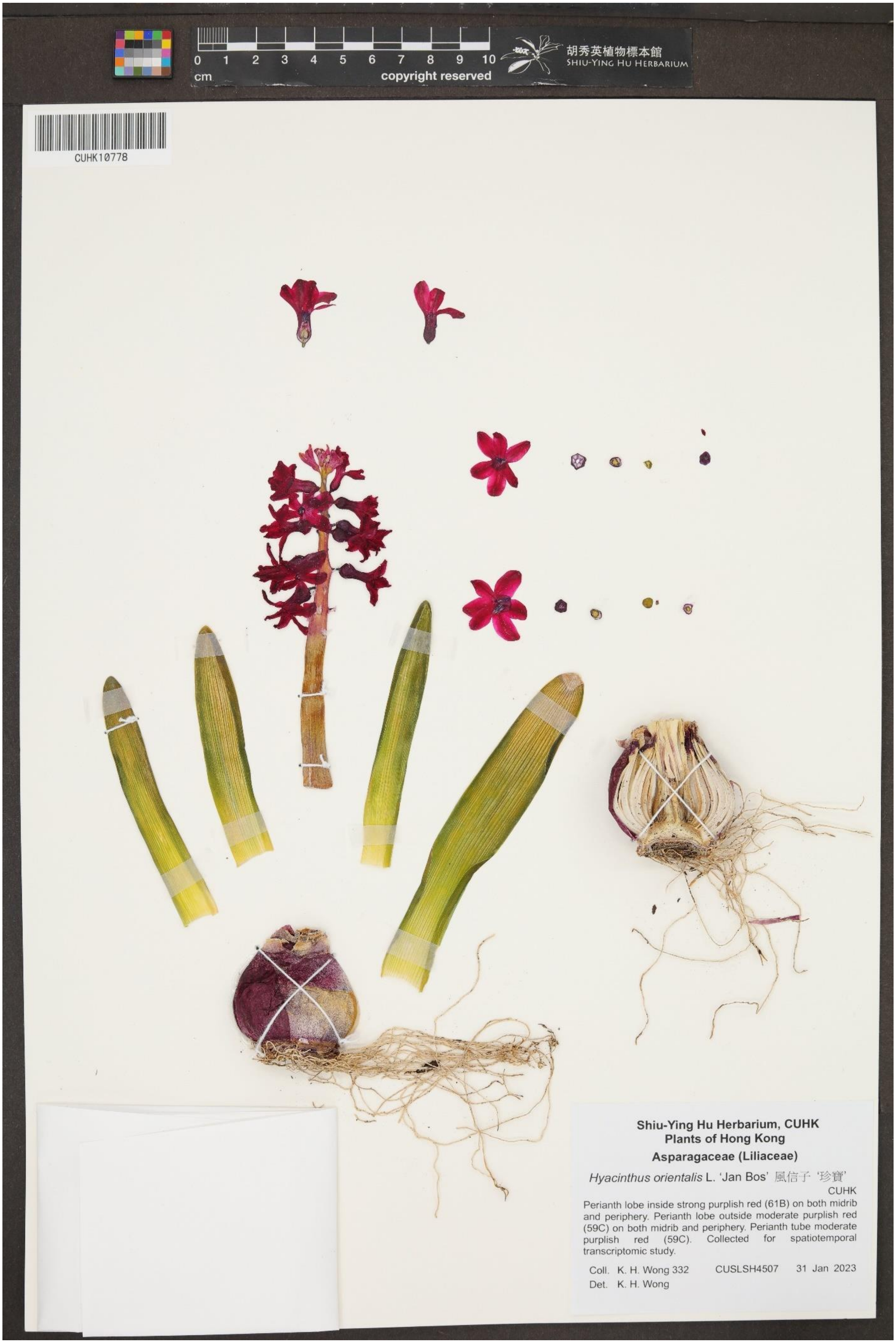

Fig. S3 – Voucher specimen of *Hyacinthus orientalis* L. 'Pink Pearl' (K. H. Wong 328)

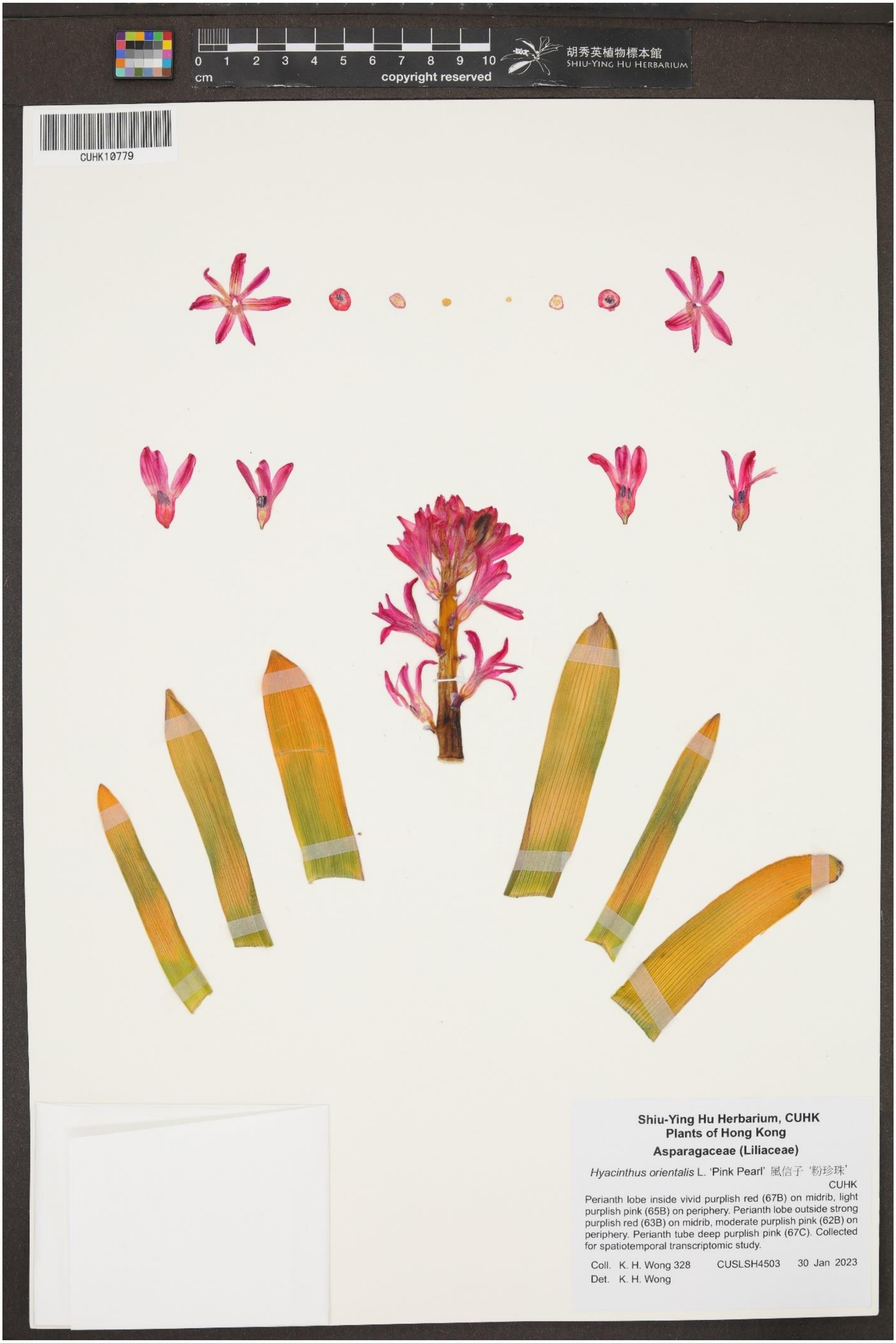

Fig. S4 – Voucher specimen of *Hyacinthus orientalis* L. ‘Gipsy Queen’ (K. H. Wong 327)

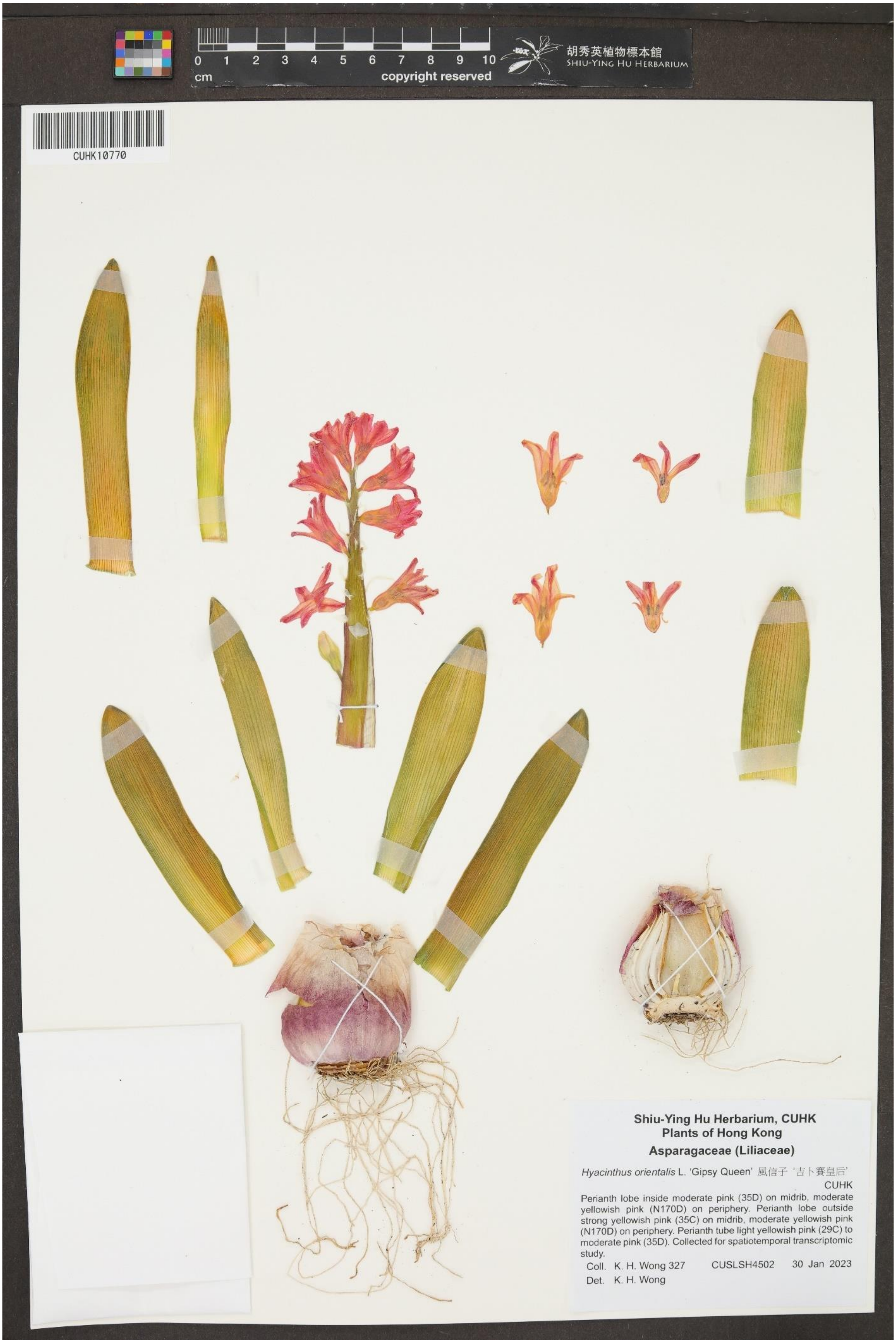

Fig. S5 – Voucher specimen of *Hyacinthus orientalis* L. ‘City of Haarlem’ (K. H. Wong 336)

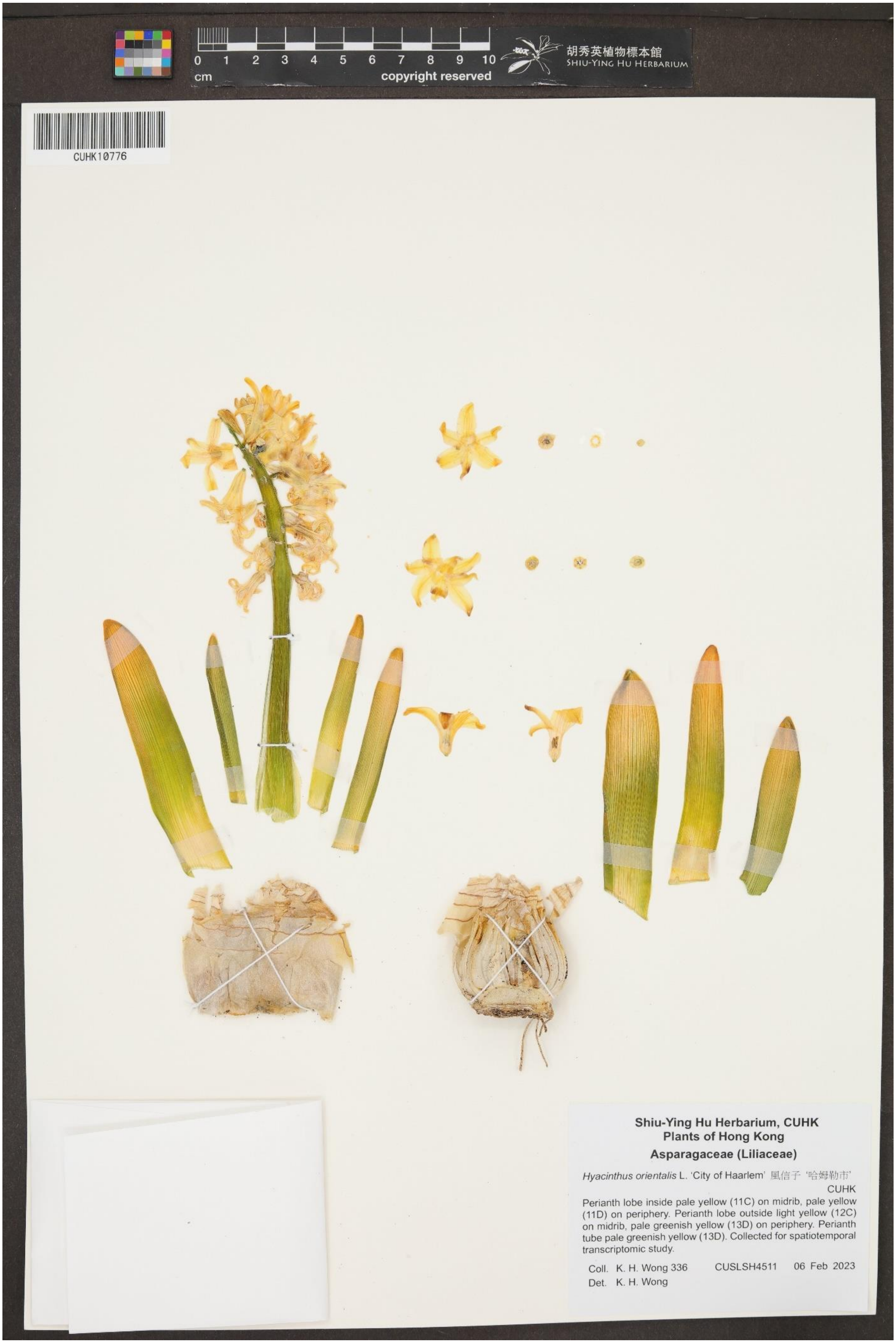

Fig. S6 – Voucher specimen of *Hyacinthus orientalis* L. 'China Pink' (K. H. Wong 333)

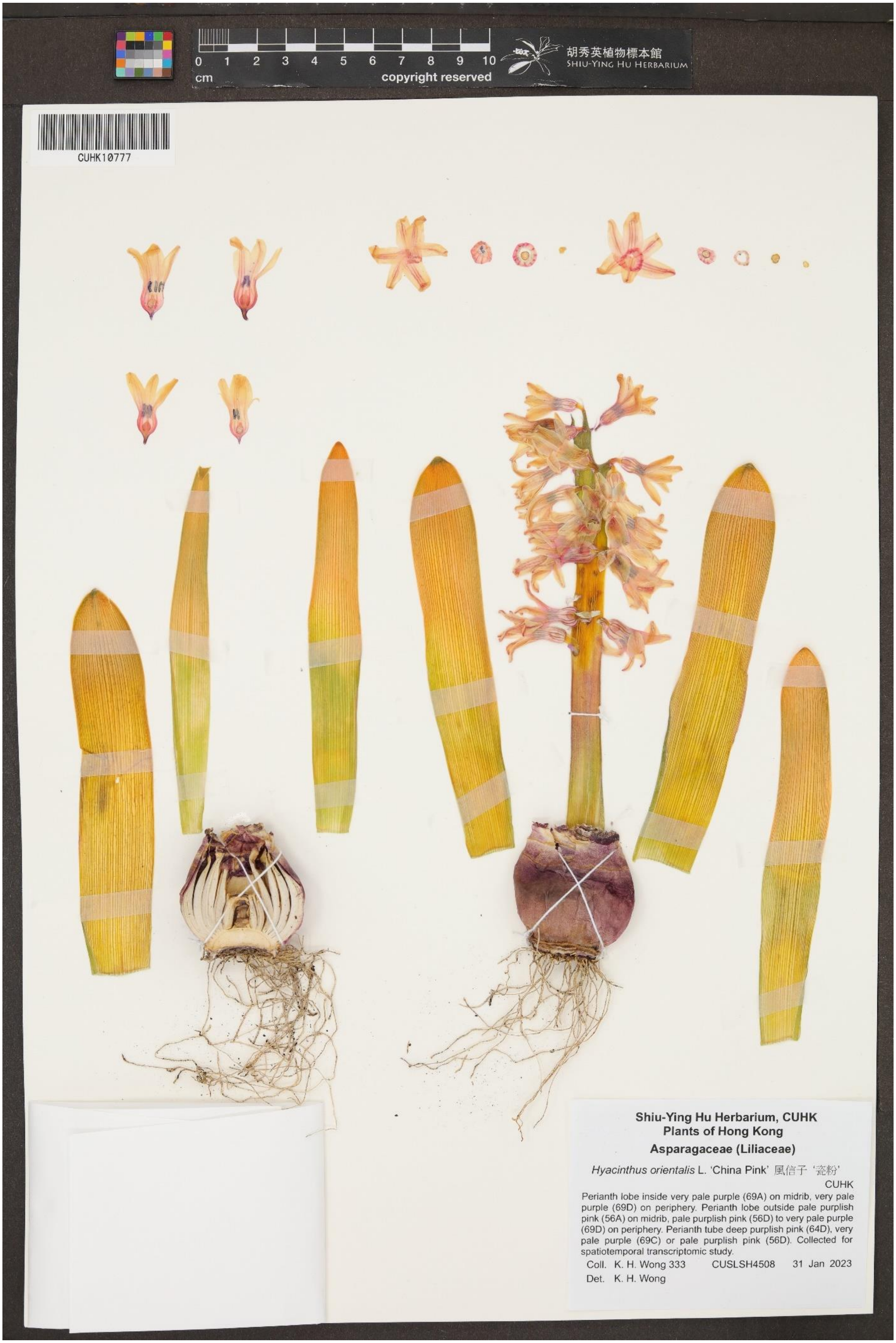

Fig. S7 – Voucher specimen of *Hyacinthus orientalis* L. 'Delft Blue' (K. H. Wong 330)

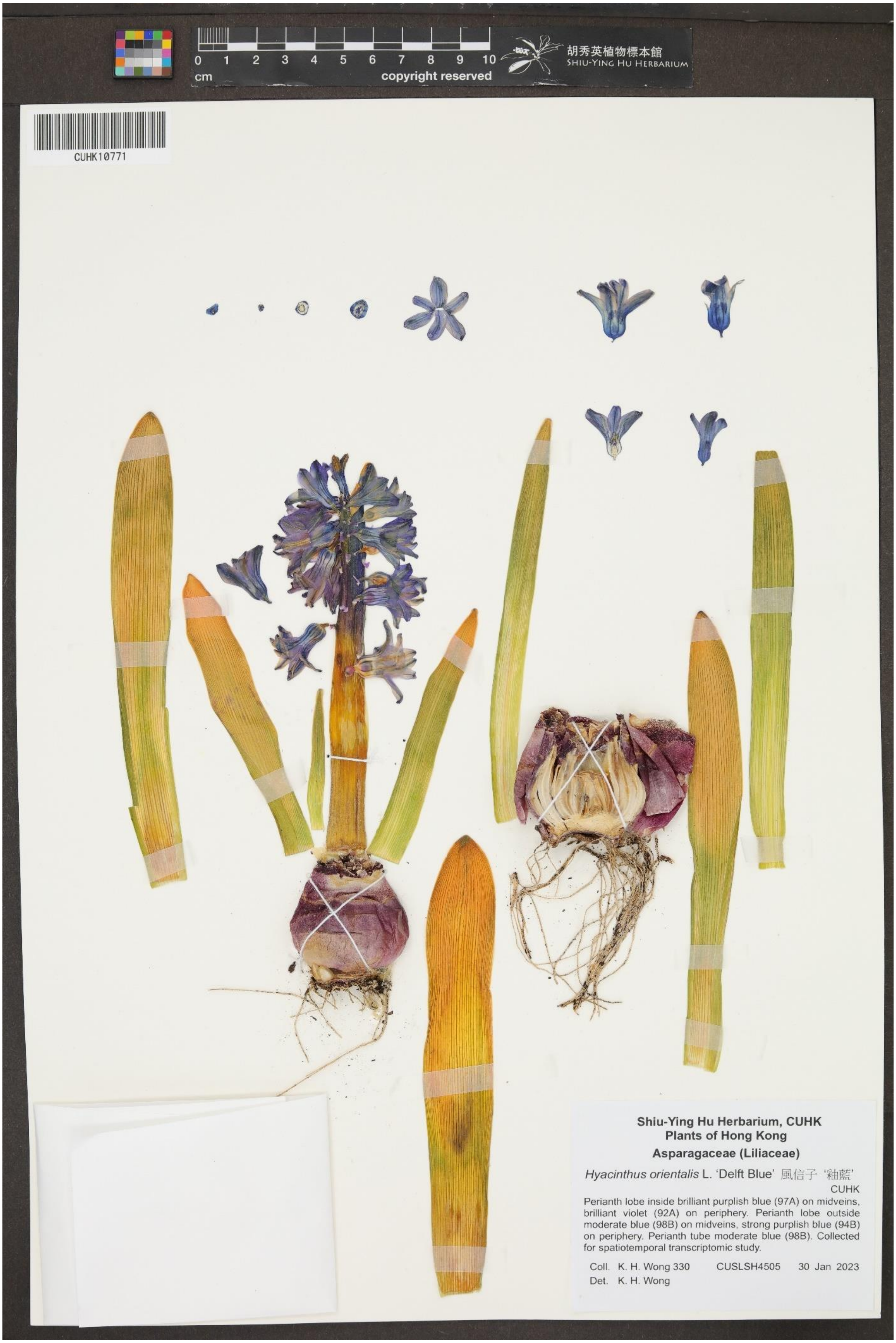

Fig. S8 – Voucher specimen of *Hyacinthus orientalis* L. ‘Peter Stuyvesant’ (K. H. Wong 335)

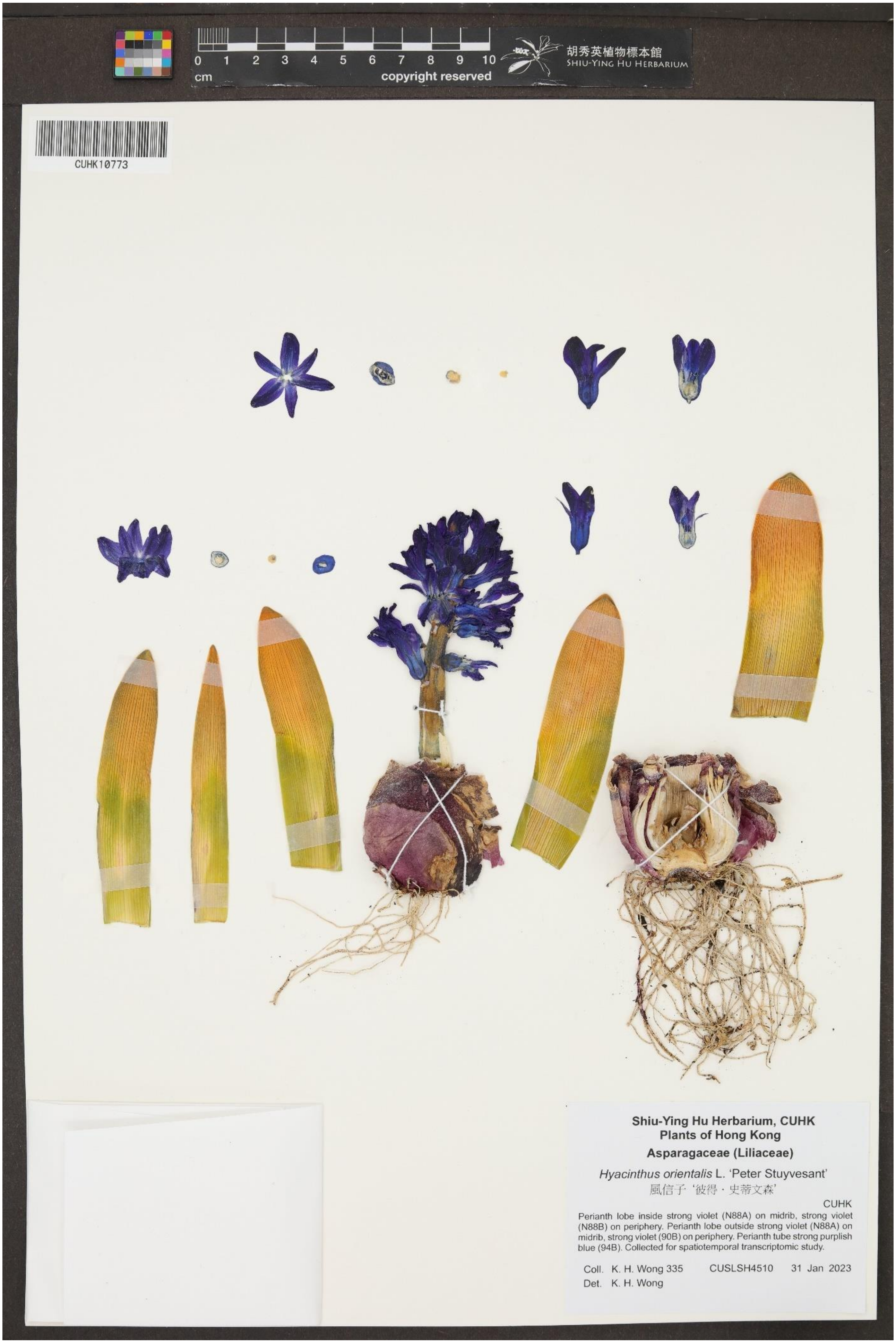

Fig. S9 – Gel record of total RNA extracted from Stage B perianth partitions

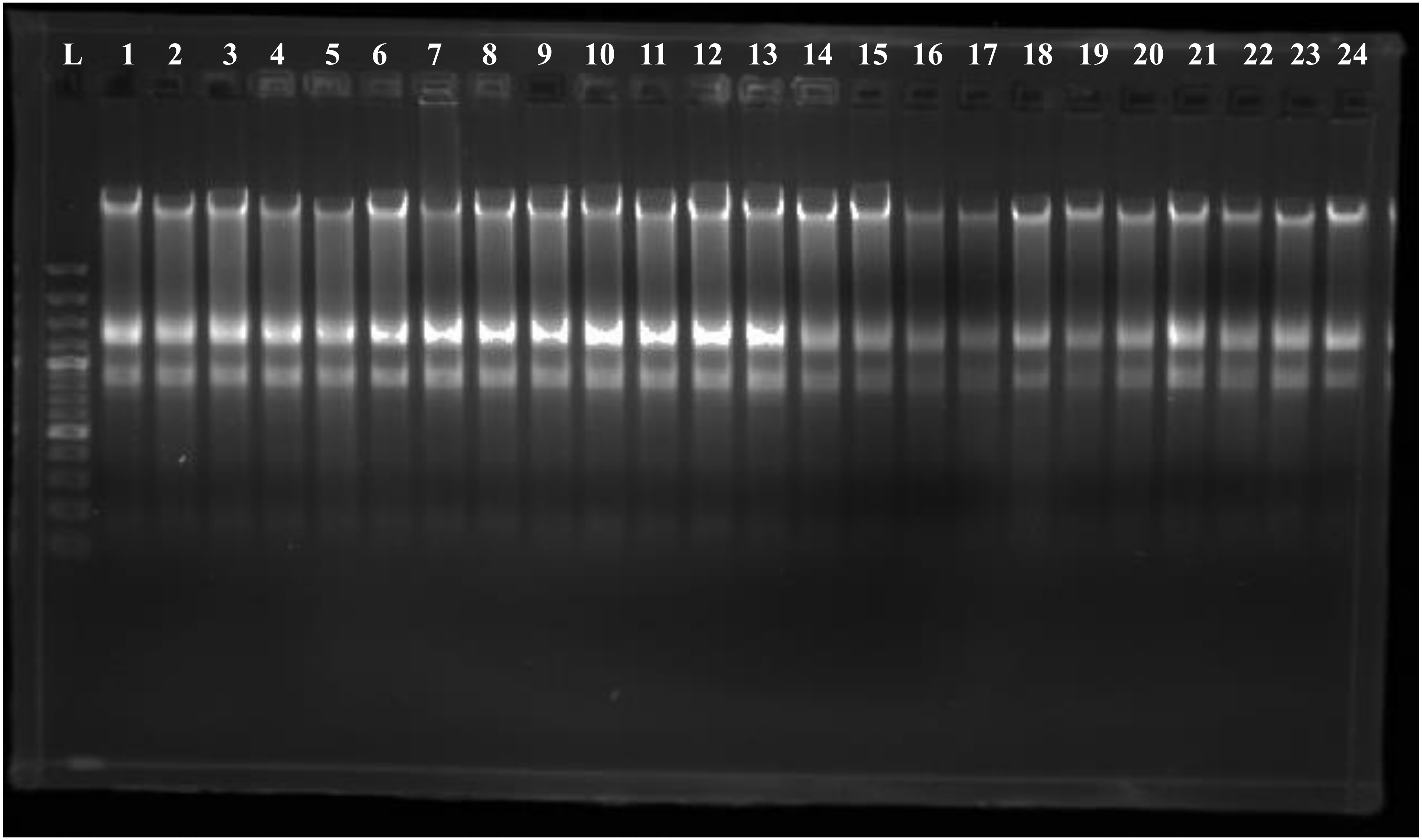

L - Ladder, 1 - CH2oB, 2 - CH2nB, 3 - CH2tB, 4 - CH3oB, 5 - CH3nB, 6 - CH3tB, 7 - DB1oB, 8 - DB1nB, 9 - DB1tB, 10 - DB2oB, 11 - DB2nB, 12 - DB2tB, 13 - DB3oB, 14 - DB3nB, 15 - DB3tB, 16 - JB1oB, 17 - JB1nB, 18 - JB1tB, 19 - JB2oB, 20 - JB2nB, 21 - JB2tB, 22 - JB3oB, 23 - JB3nB, 24 - JB3tB.

Fig. S10 – Gel record of total RNA extracted from Stage B perianth partitions (Cont')

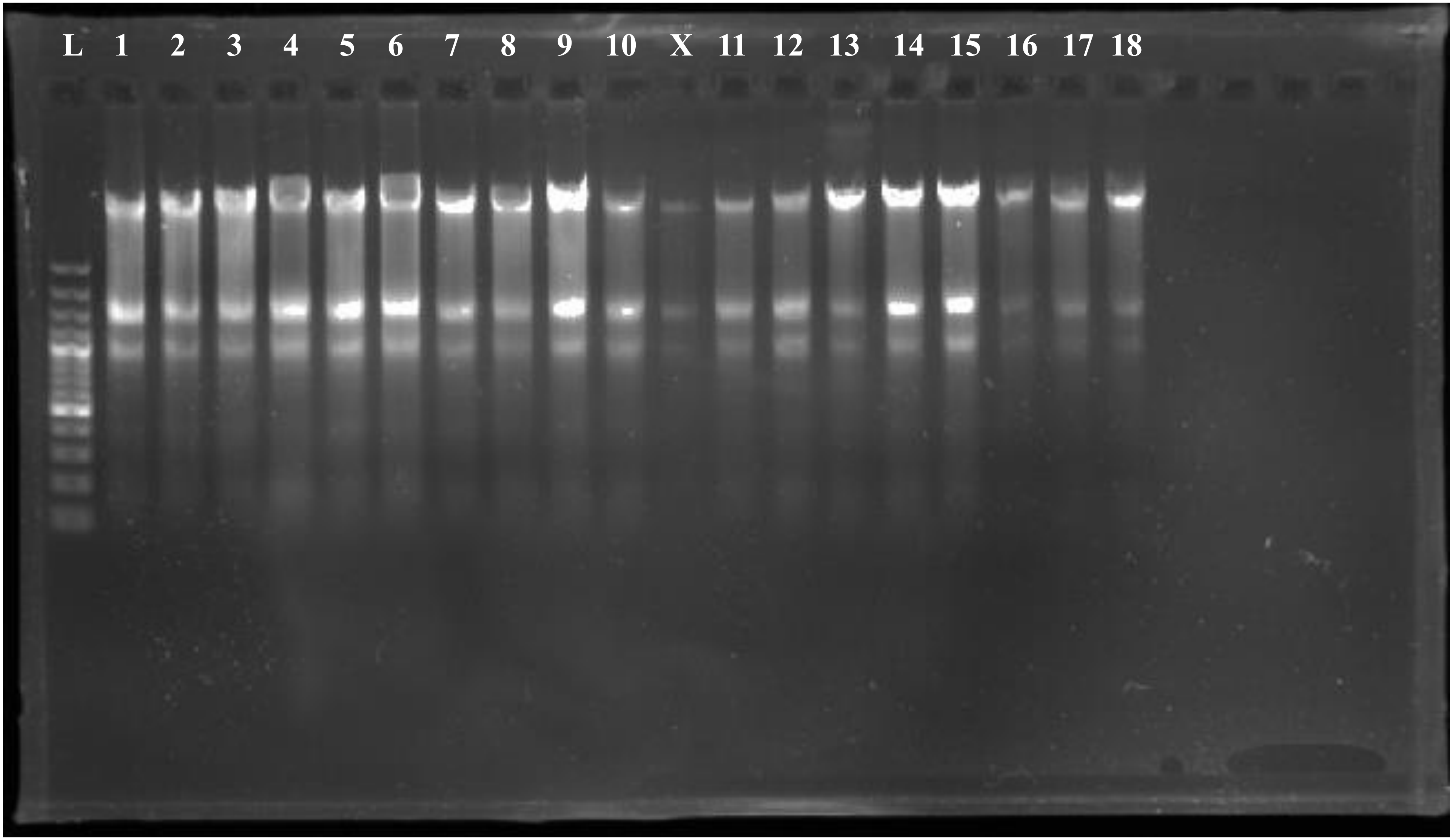

L - Ladder, 1 - CP1oB, 2 - CP1nB, 3 - CP1tB, 4 - CP2oB, 5 - CP2nB, 6 - CP2tB, 7 - CP3oB, 8 - CP3nB, 9 - CP3tB, 10 - PP1oB, X - erroneous pipetting, 11 - PP1nB, 12 - PP1tB, 13 - PP2oB, 14 - PP2nB, 15 - PP2tB, 16 - PP3oB, 17 - PP3nB, 18 - PP3tB.

Fig. S11 – Gel record of total RNA extracted from Stage B perianth partitions (Cont')

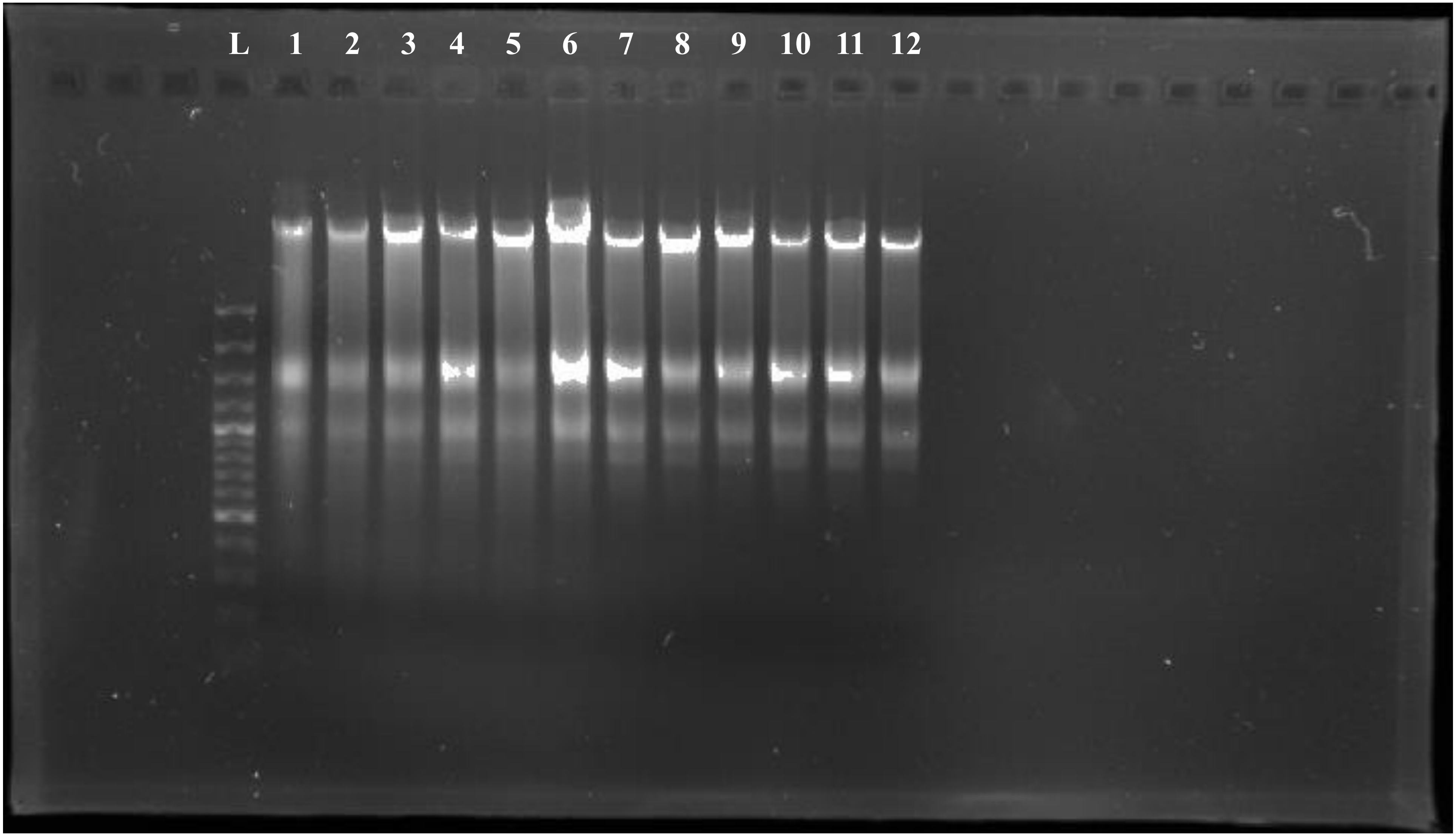

L - Ladder, 1 - GQ3oB, 2 -GQ3nB, 3 - GQ3tB, 4 - CH1oB, 5 - CH1nB, 6 - CH1tB, 7 - GQ1oB, 8 - GQ1nB, 9 - GQ1tB, 10 - GQ2oB, 11 - GQ2nB, 12 - GQ2tB.

Fig. S12 – Gel record of total RNA extracted from Stage B perianth partitions (Cont')

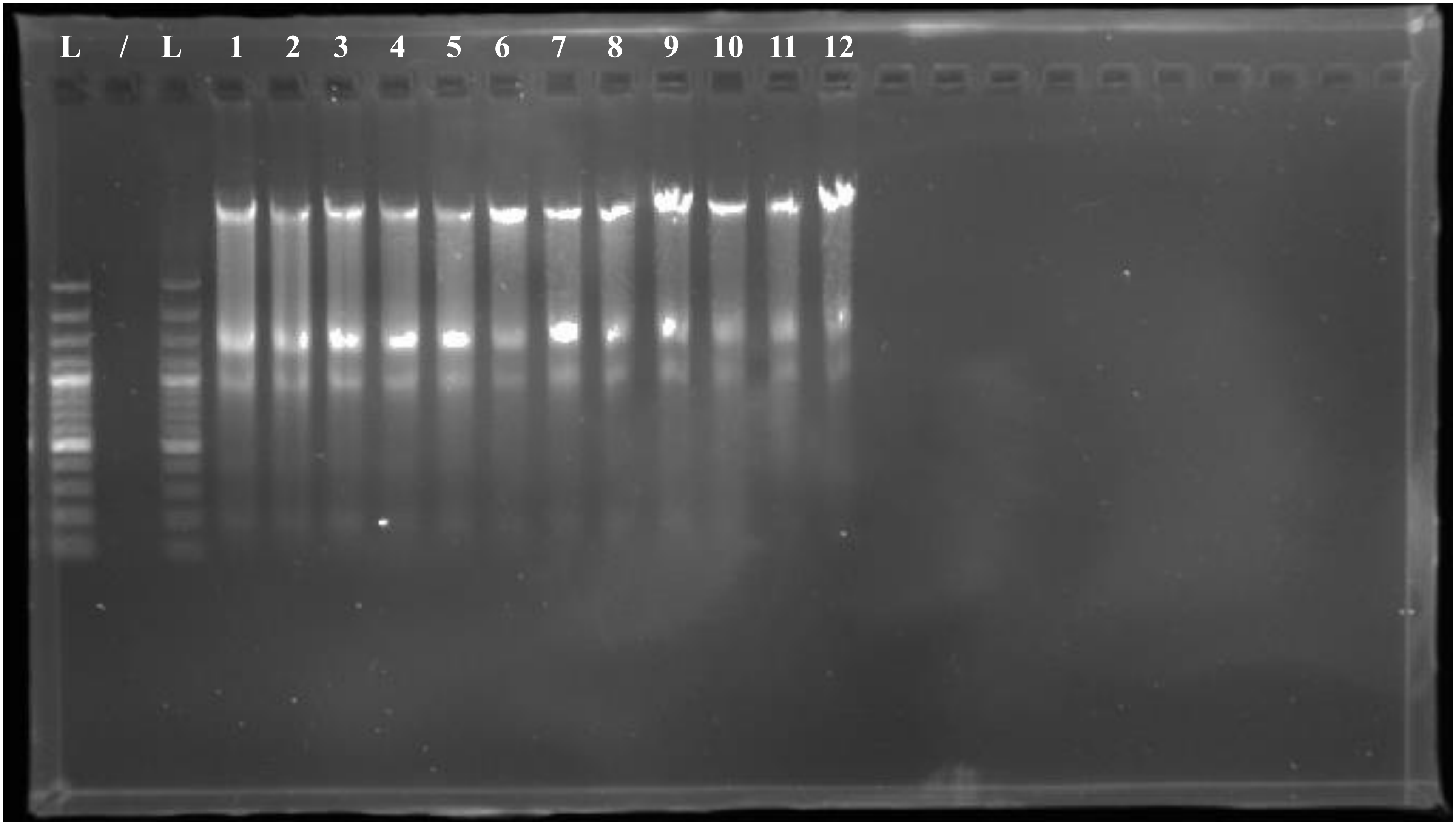

L - Ladder, / - empty, 1 - WD1oB, 2 - WD1nB, 3 - WD1tB, 4 - WD2oB, 5 - WD2nB, 6 - WD2tB, 7 - PS2oB, 8 - PS2nB, 9 - PS2tB, 10 - PS3oB, 11 - PS3nB, 12 - PS3tB. (The cultivar "WD" as 'Woodstock' was excluded from this study).

Fig. S13 – Gel record of total RNA extracted from Stage B perianth partitions (Cont')

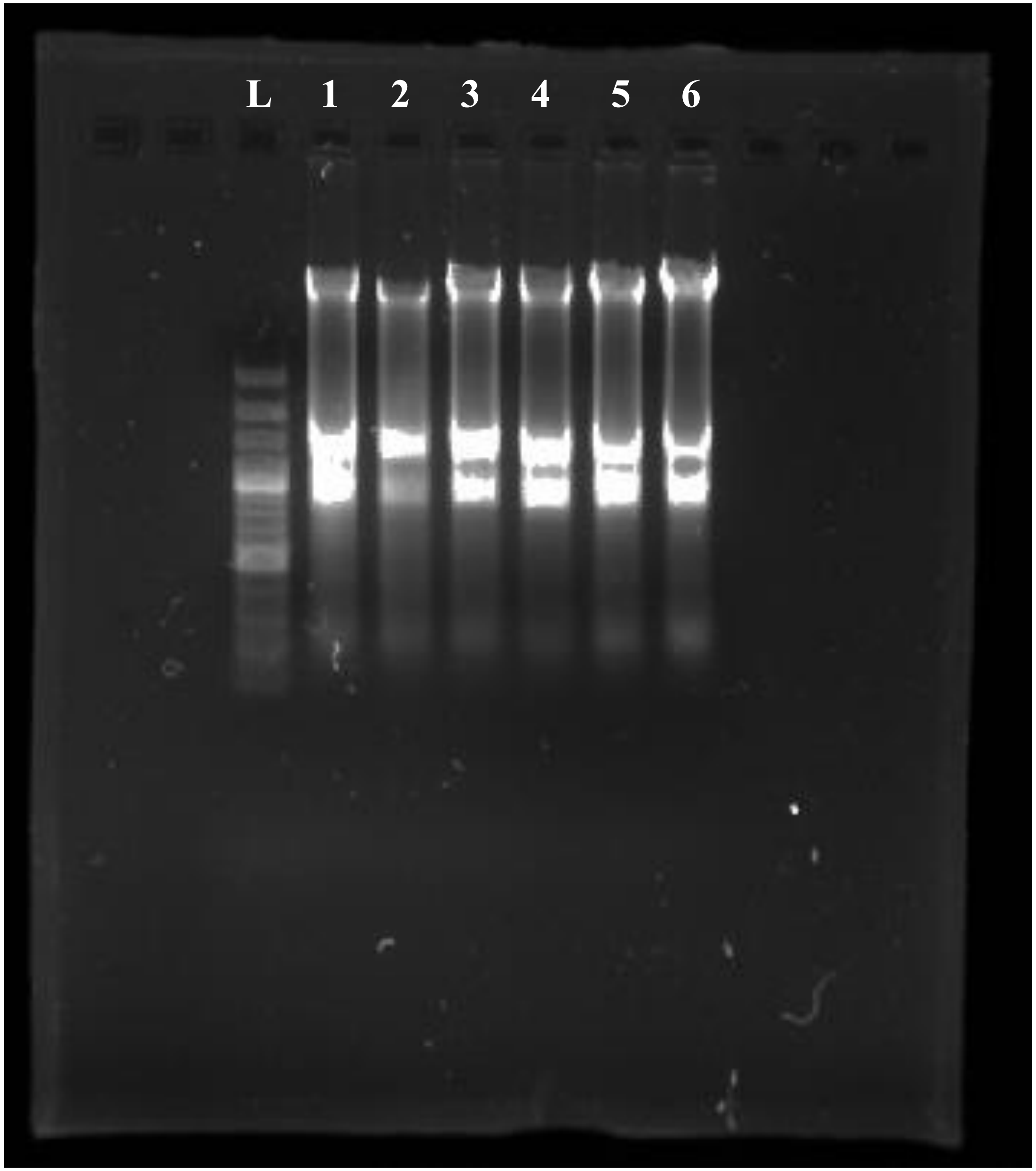

L - Ladder, 1 - WD3oB, 2 - WD3nB, 3 - WD3tB, 4 - PS1oB, 5 - PS1nB, 6 - PS1tB. (The cultivar "WD" as 'Woodstock' was excluded from this study).

Fig. S14 – Gel record of total RNA extracted from Stage C perianth partitions

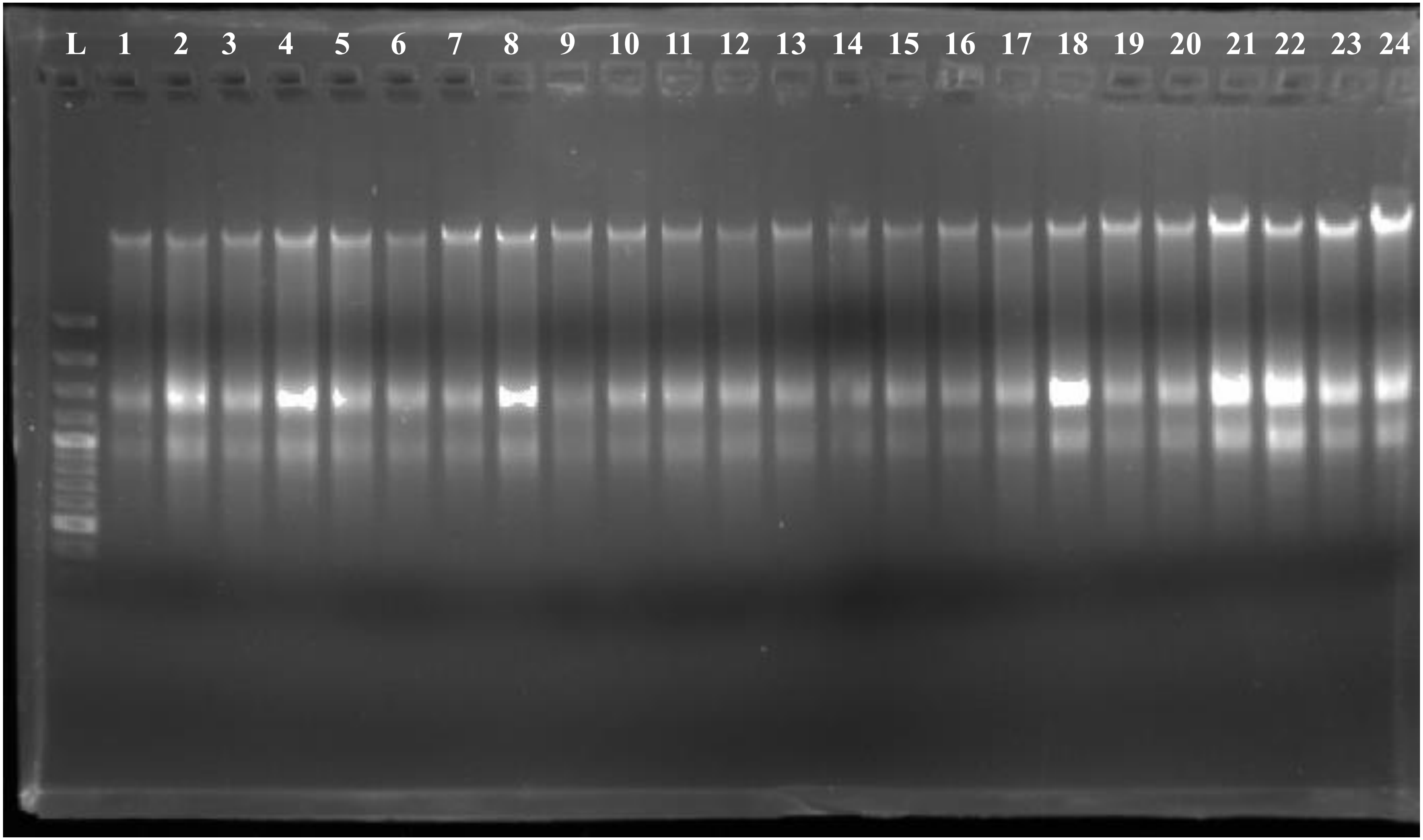

Lane L - Ladder, 1 - WD1oC, 2 - WD1nC, 3 - WD1tC, 4 - WD2oC, 5 -WD2nC, 6 -WD2tC, 7 -WD3oC, 8 - WD3nC, 9 - WD3tC, 10 - JB1oC, 11 - JB1nC, 12 - JB1tC, 13 - JB2oC, 14 - JB2nC, 15 - JB2tC, 16 - JB3oC, 17 - JB3nC, 18 - JB3tC, 19 - PS1oC, 20 - PS1nC, 21 - PS1tC, 22 - PS2oC, 23 - PS2nC, 24 - PS2tC. (The cultivar "WD" as 'Woodstock' was excluded from the study).

Fig. S15 – Gel record of total RNA extracted from Stage C perianth partitions (Cont')

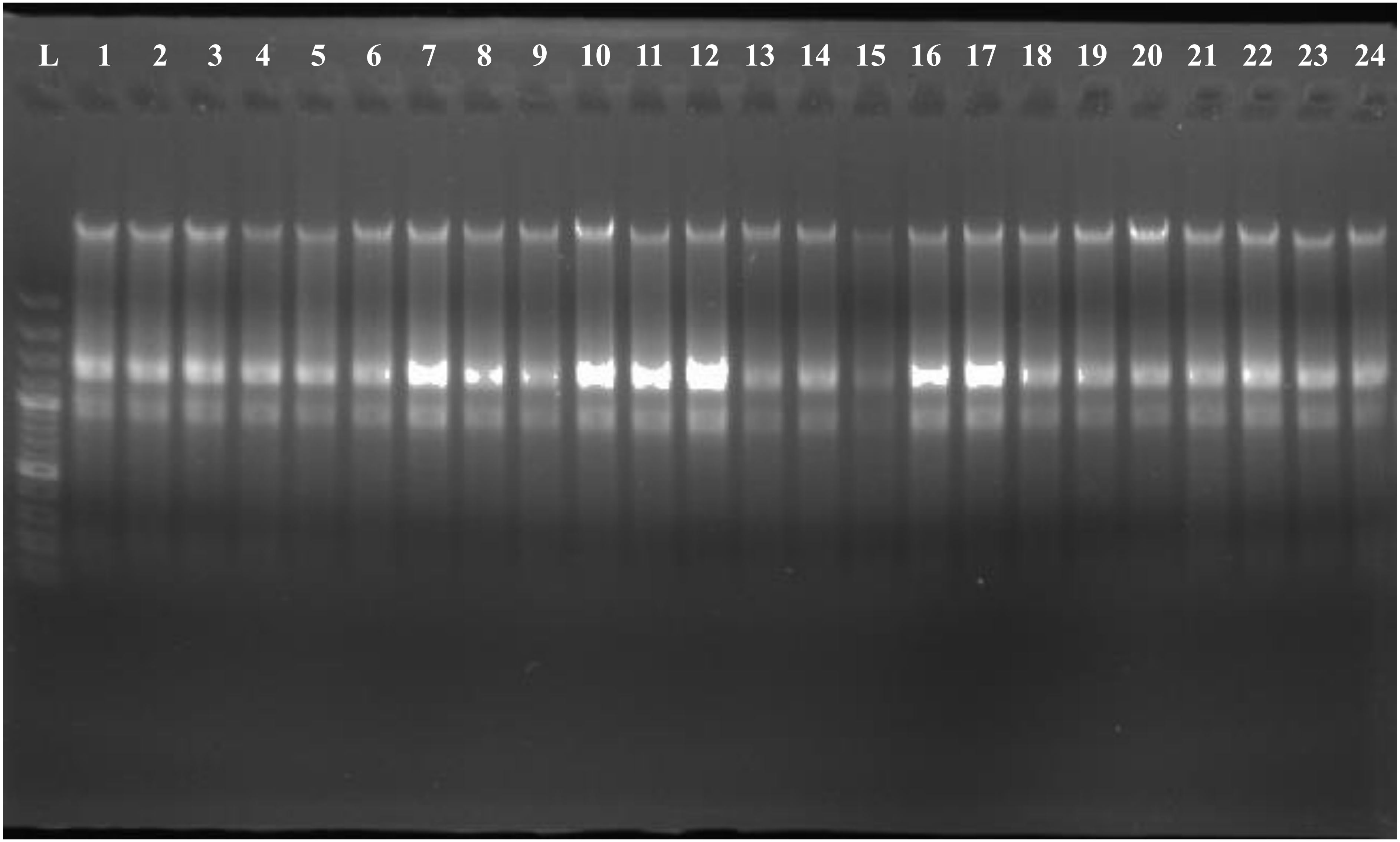

L - Ladder, 1 - PS3oC, 2 - PS3nC, 3 - PS3tC, 4 - CH1oC, 5 - CH1nC, 6 - CH1tC, 7 - CH2oC, 8 - CH2nC, 9 - CH2tC, 10 - CH3oC, 11 - CH3nC, 12 - CH3tC, 13 - PP1oC, 14 - PP1nC, 15 - PP1tC, 16 - DB1oC, 17 - DB1nC, 18 - DB1tC, 19 - DB2oC, 20 - DB2nC, 21 - DB2tC, 22 - DB3oC, 23 - DB3nC, 24 - DB3tC.

Fig. S16 – Gel record of total RNA extracted from Stage C perianth partitions (Cont')

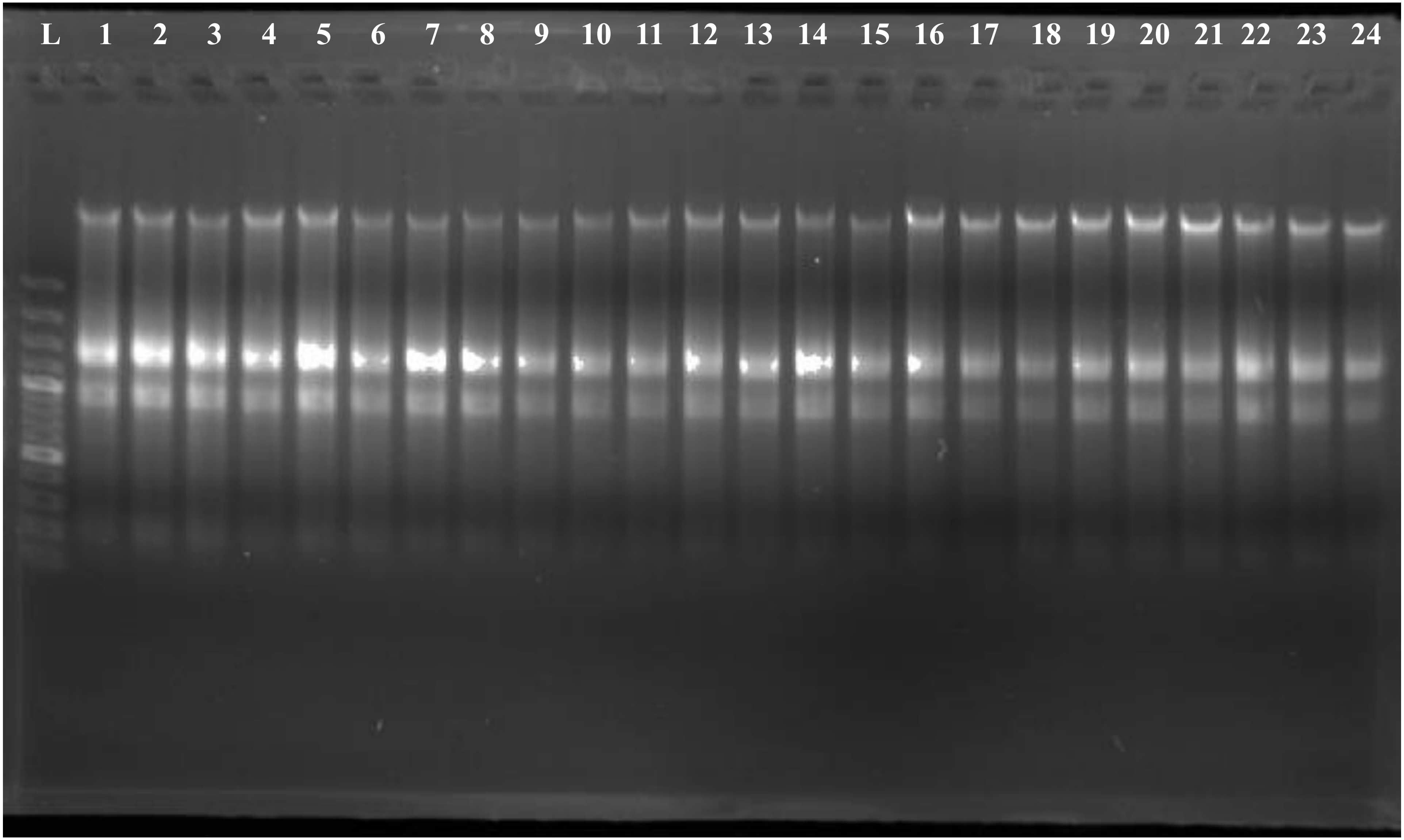

L - Ladder, 1 - PP2oC, 2 - PP2nC, 3 -PP2tC, 4 - PP3oC, 5 - PP3nC, 6 - PP3tC, 7 - GQ1oC, 8 - GQ1nC, 9 - GQ1tC, 10 - GQ2oC, 11 - GQ2nC, 12 - GQ2tC, 13 - GQ3oC, 14 - GQ3nC, 15 - GQ3tC, 16 - CP1oC, 17 - CP1nC, 18 - CP1tC, 19 - CP2oC, 20 - CP2nC, 21 - CP2tC, 22 - CP3oC, 23 - CP3nC, 24 -CP3tC.

Fig. S17 – Gel record of total RNA extracted from Stage A perianth partitions

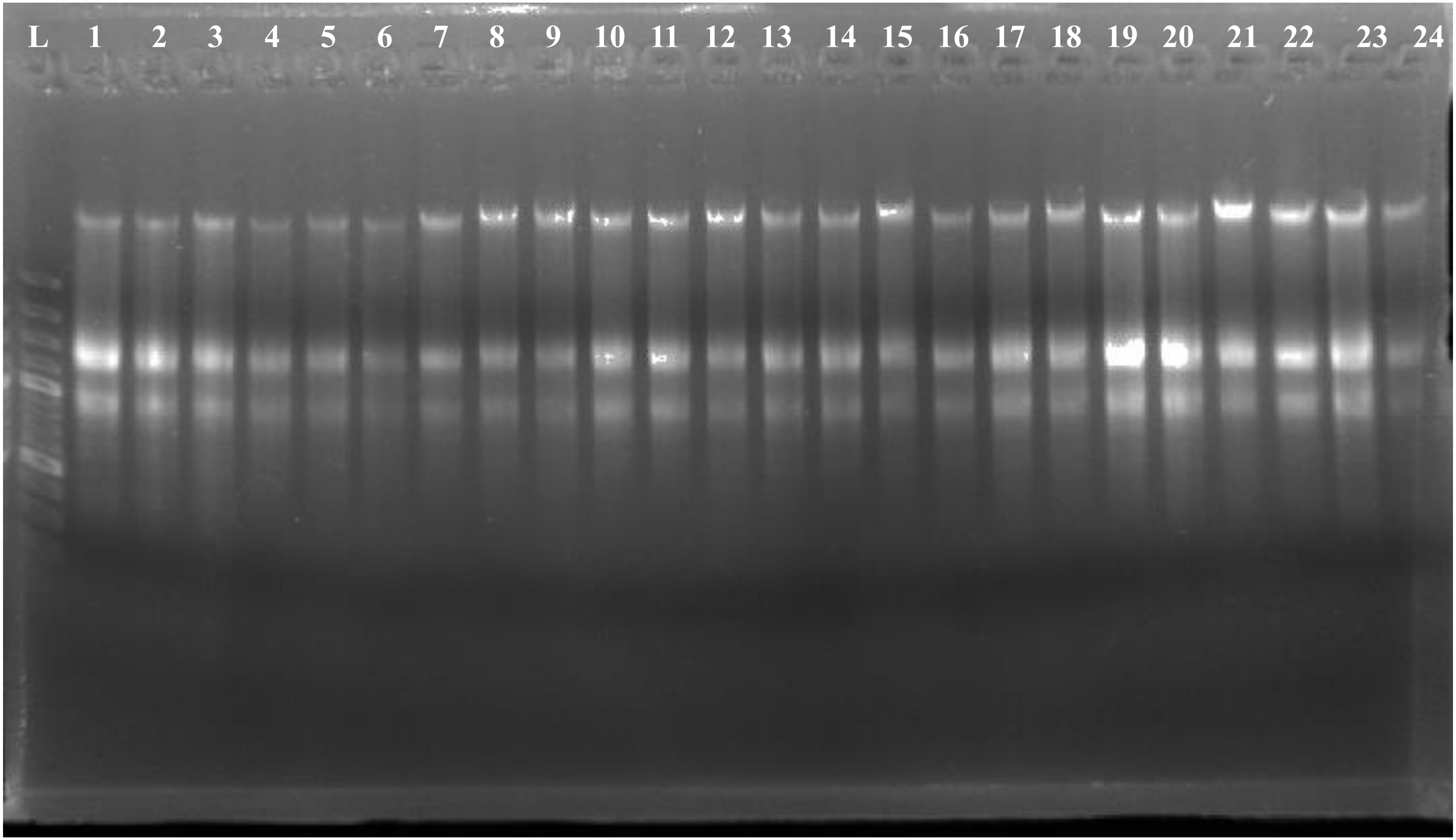

L - Ladder, 1 - GQ1oA, 2- GQ1nA, 3 - GQ1tA, 4 - GQ2oA, 5 - GQ2nA, 6 - GQ2tA, 7 - GQ3oA, 8 - GQ3nA, 9 - GQ3tA, 10 - CH1oA, 11 - CH1nA, 12 - CH1tA, 13 - CH2oA, 14 - CH2nA, 15 - CH2tA, 16- CH3oA, 17 - CH3nA, 18 - CH3tA, 19 - WD1oA, 20 - WD1nA, 21 - WD1tA, 22 - WD2oA, 23 - WD2nA, 24 - WD2tA. (The cultivar "WD" as 'Woodstock' was excluded from this study).

Fig. S18 – Gel record of total RNA extracted from Stage A perianth partitions (Cont')

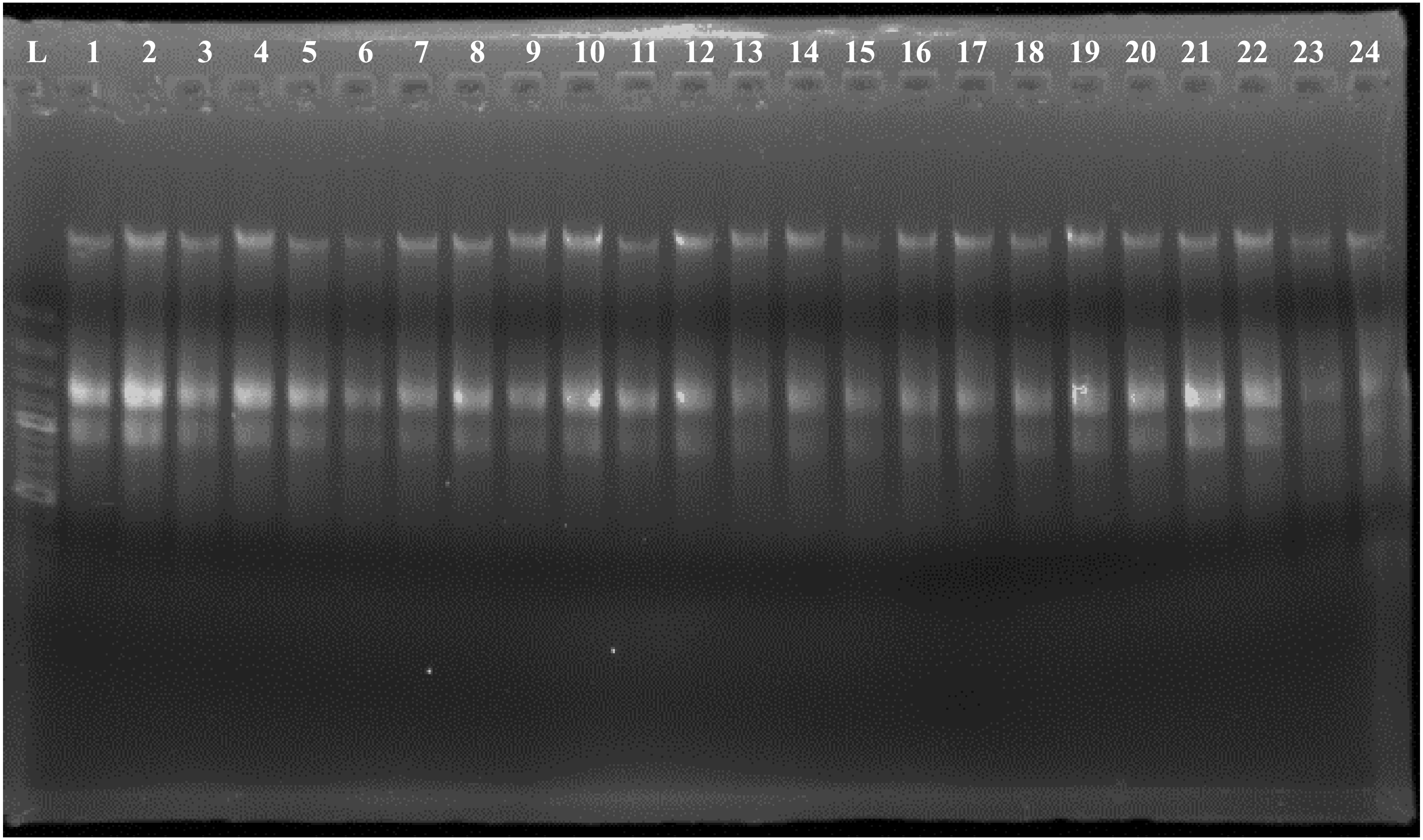

L - Ladder , 1 - WD3oA, 2 - WD3nA, 3 - WD3tA, 4 - JB1oA, 5 - JB1nA, 6 -JB1tA, 7 - JB2oA, 8 - JB2nA, 9 - JB2tA, 10 - JB3oA, 11 - JB3nA, 12 - JB3tA, 13 - PP1oA, 14 - PP1nA, 15 - PP1tA, 16 - PP2oA, 17 - PP2nA, 18 - PP2tA, 19 - PP3oA, 20 - PP3nA, 21 - PP3tA, 22 - CP1oA, 23 - CP1nA, 24 - CP1tA. (The cultivar "WD" as 'Woodstock' was excluded from this study).

Fig. S19 – Gel record of total RNA extracted from Stage A perianth partitions (Cont')

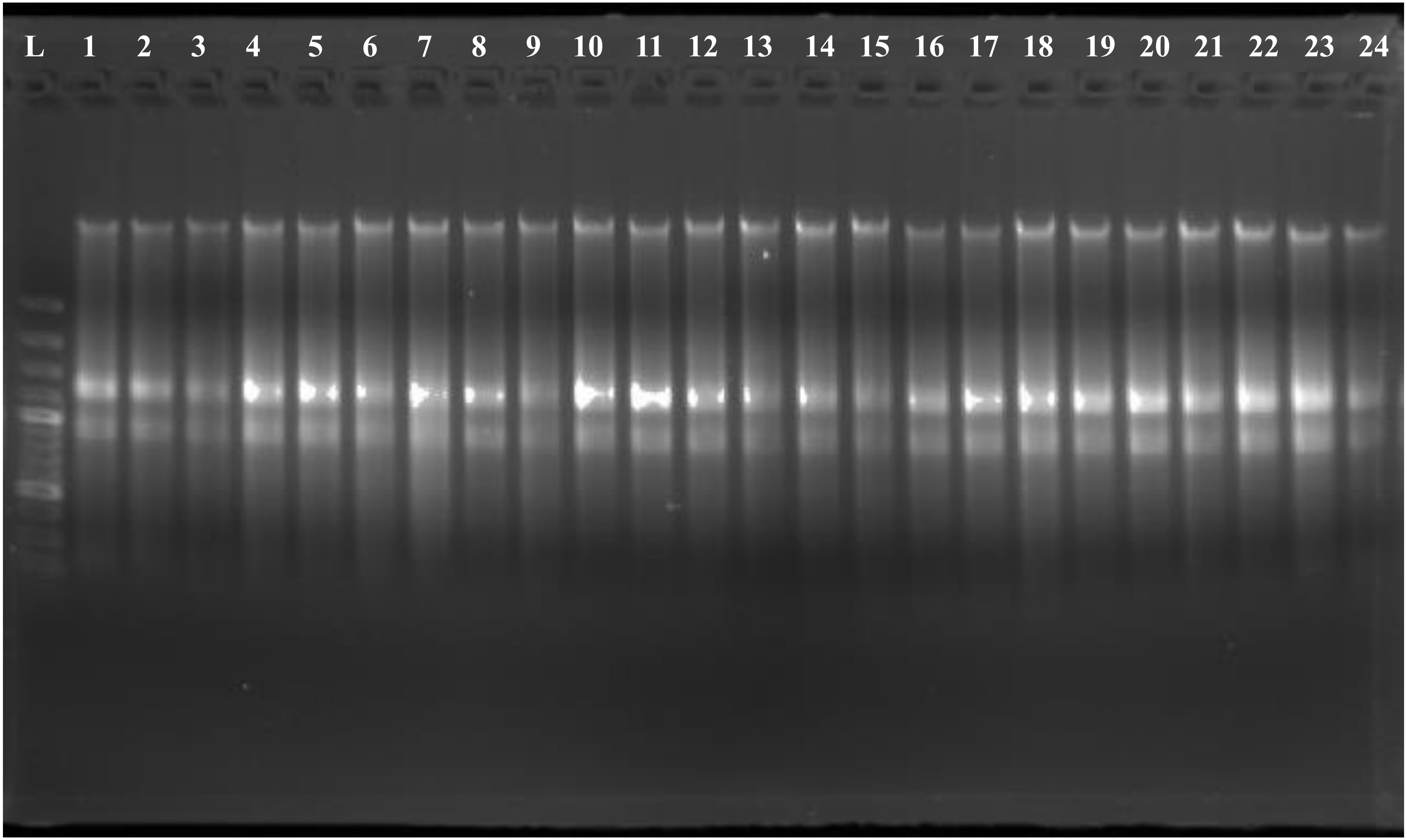

L - Ladder , 1 - CP2oA, 2 - CP2nA, 3 - CP2tA, 4 - CP3oA, 5 - CP3nA, 6 - CP3tA, 7 - DB1oA, 8 - DB1nA, 9 - DB1tA, 10 - DB2oA, 11 - DB2nA, 12 - DB2tA, 13 - DB3oA, 14 - DB3nA, 15 - DB3tA, 16 - PS1oA, 17 - PS1nA, 18 - PS1tA, 19 - PS2oA, 20 - PS2nA, 21 - PS2tA, 22 - PS3oA, 23 - PS3nA, 24 - PS3tA.

Fig. S20 – Heatmap visualisation on the top 50 DEGs across the seven cultivars at different developmental stages

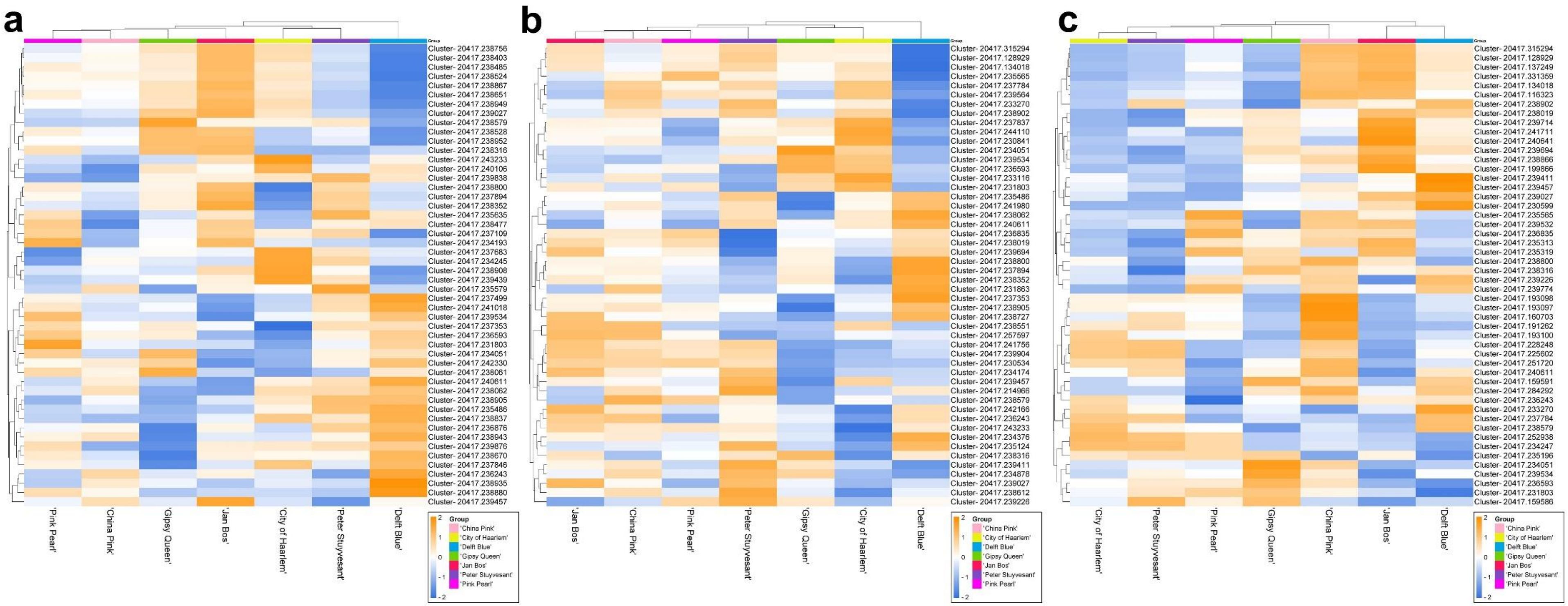

Boxes in orange represent upregulated DEGs and those in blue represent downregulated DEGs. The expression patterns of the selected top 50 DEGs are visualised for (a) green bud stage (Stage B), (b) coloured bud stage (Stage C) and (c) flowers in full anthesis (Stage A).

Fig. S21 – Photo documentation of anthocyanins and flavonoids extracts of the seven cultivars

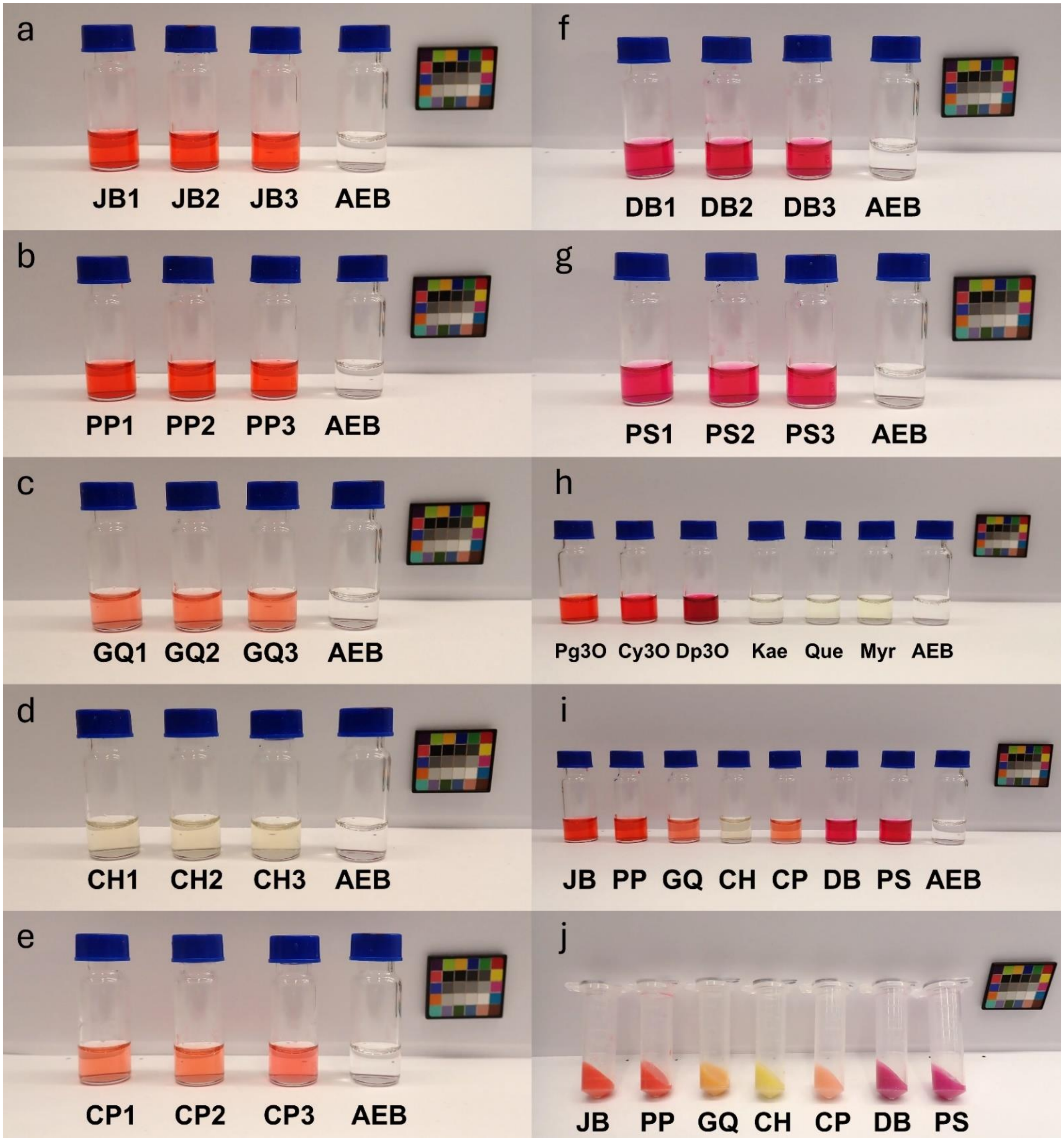

The anthocyanins and flavonoids extract of the biological replicates of (a) 'Jan Bos' (JB), (b) 'Pink Pearl' (PP), (c) 'Gipsy Queen' (GQ), (d) 'City of Haarlem' (CH), (e) 'China Pink' (CP), (f) 'Delft Blue' (DB), (g) 'Peter Stuyvesant' (PS). AEB stands for the anthocyanin extraction buffer (methanol:water:formic acid:trifluoroacetic acid in 70:27:2:1, by volume). The colour of standards of floral pigments are shown in (h). Pg3O - Pelargonidin 3-O-glucoside chloride, Cy3O - Cyanidin-3-O-glucoside chloride, Dp3O - Delphinidin-3-O-glucoside chloride, Kae – Kaempferol, Que – Quercetin, Myr – Myricetin. They were purchased from Shanghai Yuanye Bio-Technology Co., Ltd (Shanghai, China). The extracts of the seven studied cultivars are collectively shown in (i). The cell debris remains as pellets after centrifugation of the seven studied cultivars are collectively shown in (j).
